# Lung Ultrasound in Children: Principles, Techniques, and Clinical Applications

**DOI:** 10.3390/diagnostics16193079

**Published:** 2026-09-22

**Authors:** Lorenzo Di Sarno, Anya Caroselli, Valeria Pansini, Lavinia Capossela, Serena Ferretti, Anna Clemente, Anna Maria Musolino, Alberto Villani, Antonio Chiaretti, Antonio Gatto

**Affiliations:** 1 Institute of Pediatrics, Fondazione Policlinico Universitario A. Gemelli IRCCS, 00168 Rome, Italy; lorenzo.disarno1@guest.policlinicogemelli.it (L.D.S.); pansini.valeria@gmail.com (V.P.); laviniacapossela@gmail.com (L.C.); antonio.gatto@policlinicogemelli.it (A.G.); 2 Institute of Pediatrics, Fondazione Policlinico Universitario A. Gemelli IRCCS, Università Cattolica del Sacro Cuore, 00168 Rome, Italy; 3 Department of Pediatric Emergency Medicine, Bambino Gesù Children’s Hospital, IRCCS, 00165 Rome, Italy; anna.clemente@opbg.net (A.C.); amcaterina.musolino@opbg.net (A.M.M.); alberto.villani@opbg.net (A.V.)

**Keywords:** lung ultrasound, point-of-care, children, pediatrics, respiratory conditions, radiation protection

## Abstract

Lung ultrasound (LUS) has become established as a pivotal, non-ionizing imaging modality within pediatric medicine, facilitating expedited, point-of-care evaluation of diverse respiratory pathologies while eliminating patient exposure to harmful radiation. This narrative review summarizes contemporary clinical evidence regarding LUS and discusses its current utilization within pediatric medicine, focusing on fundamental physics, acoustic artifacts, standardized scanning protocols, and primary clinical indications. LUS effectively detects and characterizes pleural effusion, pulmonary consolidation, interstitial syndromes, and pneumothorax, demonstrating diagnostic performance comparable to chest radiography across numerous clinical settings while offering highly advantageous utility within diverse point-of-care clinical environments. Educational research indicates that proficiency in LUS is attainable relatively rapidly, though mastery necessitates a defined learning curve and standardized training. Consequently, LUS serves as a powerful adjunct to physical examinations in pediatrics, enhancing diagnostic precision, guiding interventions, and facilitating sequential monitoring whilst minimizing cumulative radiation. Future pediatric-focused research remains essential to refine standardized protocols, validate quantitative assessment instruments, and integrate novel technologies into clinical practice.

## 1. Background and Aim

Lung ultrasound (LUS) is a well-established imaging modality with numerous applications in pediatrics. As a rapid, portable, and practical technique, it plays a pivotal role in supporting clinical diagnosis and guiding interventional procedures [1,2,3]. LUS offers the advantage of being a point-of-care technique, allowing adequately trained healthcare professionals to utilize it as an effective extension of the physical examination [4]. Mastery of LUS is increasingly recognized as essential for pediatric clinicians, as it enhances diagnostic accuracy, guides therapeutic decisions, and facilitates real-time monitoring of disease progression and response to treatment while minimizing children’s exposure to ionizing radiation [5]. Pediatric patients, particularly infants and young children, exhibit increased sensitivity to ionizing radiation due to the higher rate of cellular mitosis occurring during early development. This heightened cellular proliferation makes their tissues more vulnerable to the damaging effects of radiation, thereby increasing the risk of long-term adverse outcomes. Consequently, minimizing radiation exposure in this population is of paramount importance in clinical practice [4,6].

As technological progress broadens the clinical uses of LUS [7], incorporating this skill into routine pediatric practice is essential for providing current, evidence-based care. However, the available evidence is heterogeneous, and LUS’s diagnostic performance should be evaluated in light of its known technical and clinical limitations. The primary aim of this narrative review is to offer a critical, concept-driven overview of the current clinical evidence on pediatric LUS, with an emphasis on bedside applicability. The review is structured in a progressive manner, moving from the fundamental principles of LUS to its more advanced clinical applications in pediatric populations, while maintaining a didactic perspective. Additionally, it seeks to provide an overview of the principal challenges and limitations associated with LUS in children, thereby elucidating its clinical relevance and identifying areas requiring further research.

## 2. Materials and Methods

We designed a narrative review of the literature. The first part explores the theoretical principles of LUS, providing all the necessary background to feel confident with this tool. The second part presents an informative overview of different applications in pediatric pulmonary conditions, pointing out the actual challenges and future feasible research perspectives. We examined the following bibliographic electronic databases: PubMed and the Cochrane Library, from inception date until August 2026. The keywords used for the search across electronic databases were: “Lung Ultrasound” or “LUS” and “Pediatric Medicine” or “Pediatric Emergency” or “Pediatric Pneumology” or “Pediatric Pneumonia”. We included studies mainly focusing on pediatric populations, prioritizing original research articles, systematic reviews, and meta-analyses with clear clinical relevance. In selected cases, additional sources such as reference textbooks and case reports were also considered to provide complementary background information or to illustrate specific clinical or technical aspects. Studies exclusively conducted in adult populations were included only when pediatric-specific evidence was limited and were interpreted with caution. Whenever both pediatric primary studies and secondary evidence were available, original pediatric studies were preferentially used to support clinical and diagnostic statements, whereas reviews and consensus documents were mainly used for background, technical standardization, and areas in which primary pediatric evidence remains limited.

English-language publications were preferentially considered. Each selected paper was reviewed and analyzed in full text by three authors (L.D.S., A. Caroselli, and A. Clemente), and any discrepancies among them were resolved by debate. Due to the heterogeneity of the articles examined, we focused on a qualitative analysis. Given the narrative design of the review, no formal risk-of-bias assessment or quantitative synthesis was performed. Nevertheless, when interpreting the available evidence, we considered study design, sample size, patient spectrum, reference standard, operator expertise, blinding, and the degree of protocol standardization, and we explicitly highlighted important sources of heterogeneity and uncertainty.

We formally declare that no generative artificial intelligence (AI) or AI-assisted technologies were utilized in the study design, data collection, analysis, or drafting of this manuscript.

## 3. Basic Principles of Lung Ultrasound

Conventional ultrasound systems assume a constant sound propagation speed (approximately 1540 m/s), whereas in the lung, the presence of air leads to marked acoustic impedance mismatch, strong reflection, and attenuation of the ultrasound beam [8,9]. Traditionally, ultrasound evaluation of the lung was in fact hardly pursued since the predominant air content of the organ results in high acoustic impedance [10,11,12]. Alveolar air content prevents the transmission of ultrasonic beams. Until the end of the 2000s, in the manual “Harrison’s Principles of Internal Medicine”, it was stated, concerning thoracic ultrasound: “Because ultrasound energy is rapidly dissipated in air, ultrasound imaging is not useful for evaluation of the pulmonary parenchyma. However, it is helpful in the detection and localization of pleural abnormalities and is often used as a guide to placement of a needle for sampling of pleural liquid (i.e., for thoracentesis)” [13]. Therefore, it was believed that ultrasound imaging of the lungs would not be possible due to significant ultrasound wave reflection at the soft tissue-air interface, which creates an acoustic impedance mismatch. This mismatch would prevent visualization of the vascular and bronchial trees as well as the lung parenchyma [4,13]. However, thanks to the pioneering work of French intensivist Daniel Lichtenstein and subsequent investigators, it has been established that chest and LUS generate characteristic sonographic artifacts, and that specific artifact patterns correlate with clinical diagnoses and conventional radiologic findings [4,14,15]. As Musolino et al. highlighted in their review [16], LUS primarily relies on the interpretation of artifacts generated at tissue-gas interfaces, rather than providing a direct anatomical representation of lung structures. Thus, it is important to interpret artifacts within the appropriate clinical and laboratory context, since similar patterns may occur in multiple clinical scenarios and even in some healthy states, requiring critical evaluation [16].

Just at the level of the interface between soft tissue and air, it is possible to visualize the so-called “pleural line,” which appears as a horizontal hyperechoic line [12,17]. Physiologically, the pleural line is subject to movement: first and foremost, it moves with respiration. This movement is the so-called “lung sliding” [12,14]. This motion is easily seen as a shimmering or “ants marching on a log” appearance [18].

Below this level, an air-filled lung generates multiple reverberations of the pleural line that appear as horizontal, artifactual echoes termed “A-lines.” By contrast, pulmonary parenchymal consolidations and collapses can be directly visualized because the absence of alveolar air abolishes A-line formation [19].

Another key artifact associated with anomalies such as pulmonary edema, interstitial phlogosis, and pulmonary fibrosis manifests in LUS as vertical, hyperechoic artifactual lines. Originating from the pleural line and extending to the bottom of the screen, these artifacts are known as ‘B-lines’ and characteristically erase physiological A-lines [20,21,22]. The physical mechanism that generates B-lines is not fully understood [19,23]. They are non-specific findings that may be observed in several conditions, and even in certain physiologic states.

Pleural effusions can be directly visualized and examined [19]. Only subpleural parenchymal abnormalities can be seen, since the air-filled parenchyma causes acoustic impedance mismatch, as previously described, at the level of the pleural line, preventing the visualization of deep abnormalities. However, most clinically relevant consolidations and other anomalies reach the pleural line and are thus detectable via LUS [19,24].

Another subtle movement of the pleural line is the “lung pulse,” which is a periodic vertical motion synchronous with cardiac pulsations that are transmitted through the pulmonary parenchyma. The rhythmic, subtle motion of the heart’s beat being transmitted through the lung tissue is normally obscured or “overpowered” by the much larger, more dynamic motion of lung sliding [25]. It indicates loss of ventilation when detected, which is consequently accompanied by an absence of lung sliding [26]. It is considered an early, specific sign of complete atelectasis, but it is also associated with selective intubation or apnea [25,27,28,29]. Furthermore, the detection of this sign confirms an intact pleural interface and excludes the possibility of pneumothorax (PTX) in the examined location [28]. This phenomenon is more easily observed in areas closer to the heart [27].

Bone can constitute a further obstacle for ultrasound investigation since it can’t be crossed by the ultrasound beam. Thus, the image produced below a rib is a shadow cone that hides the underlying area, including the pleural surface [9]. However, an oblique scan can be obtained by turning the probe along the intercostal space to expand the view of the pleura and lung. Furthermore, it is estimated that approximately 70% of the pleural surface is accessible for ultrasonic visualization. This constraint inherently limits the evaluation of the underlying parenchyma due to the acoustic shadowing produced by the thoracic cage [30,31]. Even a limited scan of the lung surface is useful for the diagnosis of diffuse pleural and sub-pleural diseases, especially in emergency and critical care settings [9,32].

All of the aforementioned findings are acquired using B-mode, which produces two-dimensional images primarily employed for the assessment of structural features. In contrast, M-mode is an imaging modality that records motion along a single ultrasound scan line, displaying time on the x-axis and depth on the y-axis. This mode is particularly valuable for evaluating pleural dynamics and diagnosing PTX [33].

Despite notable limitations—such as the inability to provide a complete panoramic view of the lungs due to the inaccessibility of the subscapular and mediastinal regions—LUS remains a highly valuable diagnostic tool. Indeed, pulmonary lesions are detectable only if they reach the pleural line; even a few millimeters of air interposed between the pleura and a pathological area can completely obscure abnormalities, regardless of their size [34]. Nonetheless, the utility of LUS does not rely solely on the direct visualization of parenchyma, but rather on the identification of specific ultrasound artifacts which enable the diagnosis of various pulmonary conditions [10,35,36].

In neonates and infants, the thin subcutaneous tissues, the relatively unossified thoracic cage, and the prominent thymus collectively serve as acoustic windows that substantially enhance the utility of LUS. The principal acoustic windows include the sternal and costochondral cartilages for anterior thoracic imaging, the intercostal spaces for evaluation of the lungs, pleura, and posterior mediastinum, and abdominal organs such as the liver, spleen, and fluid-filled stomach for assessment of the inferior thoracic cavity [37,38]. Intercostal scanning offers broad access to thoracic structures [39,40,41,42], while abdominal windows support visualization of the lower thoracic cavity [43]. Unossified sternal and costochondral cartilages typically appear hypoechoic and may vary in shape, occasionally resembling abnormal chest wall masses [44]. As children grow, progressive ossification of these structures reduces acoustic accessibility [44]. The thymus, proportionally largest during infancy, facilitates sonographic assessment of the anterior mediastinum, particularly via sternal, suprasternal, or supraclavicular approaches [38,45]. It is usually situated in the anterior mediastinum but sometimes extends into other compartments and shows variable shape, size, and symmetry that change with age and health [46,47]. It is proportionally large at birth, peaks around 4–6 months, and involutes through childhood, though it remains detectable by ultrasound even when too small for chest X-ray (CXR) [45,48]. On imaging, the thymus may mimic cardiomegaly or pulmonary/mediastinal disease on radiographs, and lung consolidation—particularly pneumonia—on ultrasound [46,47,49]. Sonographically, it appears hypoechoic with bright internal echoes and punctate foci corresponding to septa and vessels [47,50], as shown in Figure 1.

Moreover, in healthy newborns and infants, LUS patterns differ from those in older children, particularly in the first 3 months of life. During this period, multiple or confluent B-lines may be physiologic and should be interpreted cautiously within the clinical context, while pleural line abnormalities, effusions, lung points, or subpleural consolidations are always pathologic [51].

## 4. Equipment and Technique

### 4.1. Probe Choice

Contemporary ultrasound machines are generally equipped with three principal types of transducers—linear, convex, and phased-array—all of which can serve key functions in LUS [52]. Although less commonly available, some institutions may also utilize a micro-convex probe specifically designed to optimize thoracic imaging, especially in NICU settings [52,53,54].

Each of these probes offers distinct advantages in LUS, with selection guided by the clinical scenario and the anatomical area being assessed [55,56].

High-frequency linear probes (6–12 MHz) are optimal for evaluating superficial thoracic structures, providing detailed visualization of the pleural line, although their depth penetration is limited [52,53]. In newborns and young infants, high-frequency linear probes are optimal for accurate pleural and sub-pleural assessment due to their distinct thoracic anatomy, which features thinner subcutaneous tissue, reduced muscle and fat mass, cartilaginous ribs, and smaller lung volumes [28,57,58]. Convex probes may be considered in this population if a linear transducer is unavailable, although linear probes are generally preferred due to their superior pleural resolution [58,59]. Despite this physiologic rationale, few prospective studies have directly compared the diagnostic performance of different probe types in neonatal and pediatric LUS [60]. Overall, linear probes remain the preferable modality for detecting pleural and sub-pleural abnormalities, including PTX in this age group [12,57]. High-frequency linear probes are limited in their ability to assess deeper regions of the lung. This limitation can be particularly evident when attempting to follow vertical artifacts such as B-lines into the deeper parenchyma, or when quantifying B-line patterns, which may reduce diagnostic reliability [35]. In contrast, the microconvex probe, with its small footprint and favorable balance between depth penetration and image resolution, is particularly well suited for neonatal imaging. It provides adequate visualization of both superficial and deep structures and is versatile enough to be used for scanning multiple organs, including the brain, heart, lungs, and abdomen. Due to these characteristics, it has been proposed as a potential universal probe in the neonatal population. However, further studies are needed to clarify its role in LUS compared with the more commonly used linear probes [54].

Low-frequency convex probes (typically 3–5 MHz) can be useful in evaluating deeper pulmonary regions and are well suited for pediatric patients with a larger thorax or of older age [52,53,59]. Their lower frequency allows for deeper tissue penetration, making them useful when imaging beyond the superficial pleura is required; however, this comes at the expense of detailed pleural resolution and remains limited by the interposition of healthy, aerated parenchyma.

Similarly, the phased-array probe (2.5–5 MHz), while useful for imaging deeper thoracic structures, presents challenges in accurately visualizing the pleural line, making it less suitable for detailed evaluation of superficial lung findings [52,53]. However, their lower frequency makes them preferable for assessing deeper pathologies such as pulmonary edema and consolidations [12,56]. Compared to phased-array probes, convex probes may not only offer better image interpretation accuracy, but may also detect a lower number of B-lines [61,62]. The clinical implications of these differences require further investigation in large-scale, randomized controlled trials [17].

Overall, with regard to probe selection for pediatric LUS, the available literature includes a heterogeneous mix of study designs, ranging from narrative and pictorial reviews to original observational studies, small prospective comparisons, and expert consensus statements. Most publications focus on feasibility, image quality, or operator-dependent reliability rather than on head-to-head diagnostic performance across different probe types in well-defined clinical populations. Two prospective investigations are particularly relevant in this context: Gomond-Le Goff et al. evaluated the impact of probe type and operator expertise on the interpretation reliability of LUS, reporting overall good inter-rater agreement and reliability across probes and levels of experience; however, the use of nonlinear probes by novice operators was associated with the lowest agreement and reliability [63]. Vivalda et al. subsequently performed a test-validation study of ultra-high-frequency LUS in preterm neonates, demonstrating good inter-observer agreement and reliability for this specific probe category in a vulnerable population [64]. These studies, together with earlier descriptive and consensus-based contributions, suggest that probe selection may affect both image acquisition and interpretation, but the current evidence base remains fragmented, with limited direct comparative data across age groups, clinical settings, and ultrasound platforms. Accordingly, while these findings inform practical considerations, they do not yet support definitive, universally applicable recommendations on probe choice, underscoring the need for further primary research in this area.

An overview of probe types, as well as their potential features and applications, is reported in Table 1.

### 4.2. Machine Adjustment

Machine adjustment for pediatric LUS primarily involves optimizing machine settings to maximize image quality while ensuring patient safety.

The Mechanical Index (MI) represents a fundamental safety parameter in diagnostic ultrasound, reflecting the potential for non-thermal bioeffects, particularly cavitation, which is of specific concern in LUS due to the presence of air–tissue interface [69]. In newborns, where lung tissue is more susceptible to mechanical stress and acoustic exposure, careful monitoring and adjustment of MI are essential to ensure patient safety. Current evidence supports the adoption of dedicated presets in ultrasound systems for newborns, with MI values limited to ≤0.3 [70,71]. This conservative threshold significantly reduces the risk of cavitation phenomena while maintaining adequate image quality for the detection of clinically relevant artifacts, such as B-lines and consolidations.

The implementation of pre-configured pediatric LUS presets offers an important advantage in routine clinical practice by standardizing acoustic output and minimizing operator-dependent variability [70]. Such presets should be readily accessible and require minimal manual adjustment, thereby promoting consistent adherence to safety standards across operators with varying levels of expertise. This approach aligns with the ALARA (As Low As Reasonably Achievable) principle, which remains the cornerstone of ultrasound safety, particularly in vulnerable populations such as neonates and infants [70].

In older infants and children, chest wall thickness and acoustic attenuation are generally greater than in neonates, resulting in a larger and more variable safety margin at the pleural surface. Current safety recommendations therefore do not establish a single fixed MI threshold for all pediatric age groups; instead, higher displayed MI values may be used when required to obtain adequate diagnostic images, provided that acoustic output is kept as low as reasonably achievable and adjusted according to patient size, chest wall characteristics, transducer frequency, and examination conditions [71].

Noteworthy, the diagnostic utility of LUS relies predominantly on artifact interpretation rather than high-resolution tissue imaging, allowing clinicians to operate at lower acoustic power settings without compromising diagnostic accuracy [16].

These safety-oriented adjustments reinforce the suitability of LUS for repeated bedside assessments, longitudinal monitoring, and widespread use in both acute and outpatient pediatric settings, without exposing patients to significant bioacoustic risk.

It is standard practice in pediatric LUS to set the depth so that the pleural line is centered in the image, which for neonates is typically in the range of 3–4 cm [54], and for older children, a slightly greater depth is required due to increased chest wall thickness [72].

Gain is the electronic amplification of the returning echo signals, which adjusts the overall brightness of the image without altering the underlying tissue characteristics [73]. Gain is adjusted so that the pleural line appears bright (hyperechoic), the ribs cast dark acoustic shadows, and surrounding tissues maintain appropriate gray-scale contrast without obscuring artifacts [37,74].

The focal zone in LUS denotes the segment of the ultrasound beam where the lateral resolution is at its highest, thereby producing the greatest image clarity and sharpness. Proper adjustment of the focal zone is essential, as positioning it at or near the pleural line enhances the delineation of pleural structures and improves the detection of subtle sonographic signs [37]. Machine settings should be adjusted according to the specific ultrasound system and clinical context, with particular attention to the optimization of gain and depth. Maintaining a total gain below 50% is crucial to prevent blooming artifacts, which can artificially broaden the pleural line and distort B-line morphology, potentially leading to diagnostic inaccuracies regarding pleural thickness and interstitial involvement.

Certain image processing features such as spatial compounding, harmonic imaging, and speckle reduction filters are often turned off or minimized because they can suppress key LUS artifacts like lung sliding and B-lines, which are crucial for diagnosis [75,76]. Tissue harmonic imaging, based on the detection of higher-frequency signals generated during nonlinear propagation of the ultrasound beam, may improve image quality by reducing noise and enhancing contrast resolution [77], although their impact on artifact-based imaging must be carefully considered. Some ultrasound machines may also provide software presets with lung zone labels to facilitate systematic scanning. Automated programming methods are under development to detect pleural line depth and automatically adjust MI and other parameters accordingly, further improving safety and ease of use [78].

### 4.3. Patient Positioning and Scanning Protocol in Children

LUS in children ought to follow a systematic approach based on consistent anatomical landmarks. Common reference lines include parasternal, mid-clavicular, and axillary lines. Subcostal and intercostal acoustic windows through the liver and spleen facilitate evaluation of the lung bases, while a supraclavicular approach allows assessment of the apices [34,59].

Scanning can be performed with axial, longitudinal, or oblique planes. Frequency adjustment is crucial: high-frequency probes enhance pleural evaluation (e.g., PTX), whereas lower frequencies improve visualization of deeper lesions such as consolidations or atelectasis [34].

Standardized protocols typically divide the thorax into 12 regions—6 per hemithorax—delineated by the anterior and posterior axillary lines and transverse lines above the nipples and diaphragm. These areas are further classified as anterior, lateral, or posterior [59].

An alternative method, used in newborns and younger children, divides the chest wall into six zones, delimited by the anterior and posterior axillary lines, each scanned from apex to base, with the probe held perpendicular to the ribs at each intercostal space [29].

The American Academy of Pediatrics describes a six-zone protocol for neonatal LUS, consisting of three scanning zones on each hemithorax (zone 1 is the upper lung, zone 2 is the lower lung, and zone 3 is the lateral lung) with a high-frequency linear probe, typically in the supine position [65]. However, this report does not establish this six-zone approach as the preferred or uniquely recommended neonatal LUS protocol. Neonatal LUS protocols remain heterogeneous, and pediatric approaches using 6, 10, 12, or 14 scanning regions have been described according to patient age, clinical setting, and examination purpose.

International expert consensus, such as the European Society of Intensive Care (ESICM) and the European Society of Paediatric and Neonatal Intensive Care (ESPNIC) statement, supports multi-zone (12-zone, 6 per hemithorax) protocols for quantitative LUS in pediatric patients older than 1 year [79]. This approach divides each hemithorax into six zones (anterior, lateral, and posterior regions, both upper and lower), ensuring comprehensive coverage of the lung parenchyma. The consensus highlights that this extended protocol improves the detection and quantification of lung aeration loss and pathology [79]. Simplified approaches with fewer scanning regions are, however, supported for younger patients. For example, 6 regions (3 per hemithorax) are recommended for newborns in the first 24–48 h of life, and 10 regions (5 per hemithorax) for infants and newborns older than 48 h [79]. In the first 48 h of life, the proposed protocol is simplified because gravitational effects are not yet fully established and respiratory failure is frequently related to relatively homogeneous conditions such as respiratory distress syndrome; accordingly, fewer scanning zones may be sufficient during the first 24–48 h of life, while extended protocols including posterior regions are more appropriate thereafter or in heterogeneous lung injury. In addition, the lateral lung field is generally assessed as a single zone because of the limited size of the neonatal and infant thorax.

The LUS protocol originally developed by Soldati et al. [36,80] for adult patients, which divides each hemithorax into seven zones (three posterior, two lateral, and two anterior regions, for a total of 14 scanning areas), was subsequently adapted to the pediatric setting by Di Sarno et al. [36]:

- The posterior areas should correspond to the upper, middle, and basal scans on the paravertebral line, respectively, at the spine of the scapula, the inferior angle of the scapula, and above the curtain sing (which is the air in the costophrenic recess casting a hyperechoic “air curtain” over the recess, obscuring the outline of the lateral diaphragm and moving with respiratory acts [81]);
- The lateral areas should correspond to the upper and basal scans on the midaxillary line, respectively above and below the internipple line;
- The anterior areas should correspond to the upper and basal scans on the midclavicular line, respectively above and below the internipple line.

The 14-zone approach was designed to maximize sensitivity for detecting peripheral lung pathology, and is used in both research and clinical settings for comprehensive lung assessment [82,83].

When compared to other validated protocols, such as the six-zone and twelve-zone techniques, the 14-zone method offers greater anatomical coverage but at the cost of increased examination time. Some structured scanning protocols and quantitative approaches were originally developed in adult populations and later adapted to pediatric practice; therefore, their feasibility and diagnostic yield may vary according to age, body size, and clinical setting. Table 2 provides a summary of the characteristics of the examined scanning protocols.

## 5. Ultrasonographic Basic Semiotics

LUS is fundamentally based on the interaction between ultrasound waves and the air–tissue interface. Because of the marked acoustic impedance mismatch between soft tissues and air, most of the ultrasound beam is reflected at the pleural surface, which prevents direct visualization of the lung parenchyma. Consequently, LUS primarily relies on the interpretation of artifacts generated at this interface rather than on direct anatomical imaging [16]. These artifacts form the foundation of LUS semiotics. Upon placing the probe on the thoracic wall, several key structures can be identified: the chest wall (comprising skin, subcutaneous tissue, and fat with medium-to-low echogenicity), the ribs (hyperechoic bony structures with posterior acoustic shadowing due to ultrasound absorption), the intercostal muscles (medium echogenicity), and the pleural line [9]. The pleural line appears as a thin hyperechoic structure created by the acoustic impedance difference between soft tissues and air. It does not represent a direct anatomical visualization of the pleura, but rather an interface artifact arising from the chest wall–lung boundary [84]. Indeed, the normal pleura itself cannot be directly visualized by ultrasound, and the observed hyperechoic line corresponds to the reflection at the aerated lung interface [84]. Its synchronous respiratory movement is described as the “sliding sign” or “gliding sign”; absence indicates pathology, most often PTX, but may also result from fibrothorax, bullae, or interstitial emphysema [4,85,86,87,88]. In longitudinal scans, the profile of two ribs and the pleural line between them form a shape that resembles a bat, known as the “bat sign” (represented in Figure 2) [28]. This makes it possible to confidently recognize the pleural line, even in difficult examinations.

A-lines are horizontal hyperechoic lines arising from the pleural line, parallel and equidistant from the pleura, produced by reverberation artifacts when ultrasound waves reflect repeatedly between strong interfaces like the chest wall and a normal aerated lung. They are typically seen in normal aerated lungs and help exclude pathology in the scanned area [12,14,37,38,89,90]. A-lines are represented in Figure 3.

B-lines are vertical hyperechoic artifacts arising from the pleural line and extending to the bottom of the screen, erasing A-lines. Their exact physical mechanism remains incompletely understood; current models suggest that they arise from complex acoustic interactions within peripheral lung tissue when the normal air–tissue arrangement is altered, rather than from direct visualization of interstitial thickening or fluid [20,23,37]. They have a comet-tail appearance and move with respiration [37]. Compact or coalescent B-lines can produce a “white lung” pattern, which is also known as severe alveolar–interstitial syndrome [37,90,91,92,93,94]. However, B-lines are non-specific artifacts and should not be interpreted as a direct surrogate of pulmonary edema or extravascular lung water. They may occur in a wide range of pathological conditions, including pulmonary edema, pneumonia, ARDS, atelectasis, and interstitial lung disease, as well as in certain physiological conditions [20]; therefore, their interpretation always requires critical evaluation and integration with other findings. In fact, B-lines may also reflect functional states, such as incomplete lung expansion in dependent regions of healthy infants, and are therefore not specific to any single disease [34]. The appearance and number of B-lines may also depend on technical factors, including transducer type and frequency, insonation angle, gain settings, focal characteristics, and image-processing algorithms; consequently, the number and morphology of visualized B-lines may vary according to acquisition settings [20,95]. In particular, the use of excessive gain may increase the number of visible artifacts, while the impact of tissue harmonic imaging remains debated [95]. B-lines are shown in Figure 4.

Technical factors vary with age. In newborns, high-frequency (9–14 MHz) linear probes optimize visualization of pleura, chest wall, and peripheral parenchyma, whereas lower-frequency probes are preferred in older children for deeper structures. Scanning is typically done supine in neonates, while sitting or supine positions are used in older patients [30].

Unique pediatric features also enhance acoustic access: partial ossification of the chest wall, hypoechoic costal and sternal cartilage, and the thymus all provide additional windows. As ossification progresses with age, these advantages diminish [16,40,96].

## 6. Applications in Pediatric Medicine

### 6.1. Pneumonia

Although the diagnosis of pneumonia remains primarily clinical, CXR continues to serve as the main imaging modality in suspected pediatric cases. Nevertheless, accumulating evidence indicates that LUS is a reliable and complementary diagnostic tool [4,97,98,99,100].

A meta-analysis by Pereda et al. concluded that LUS demonstrates optimal accuracy for diagnosing pneumonia in children [99]. Likewise, Orso and colleagues reported that LUS maintains good diagnostic performance across pediatric age groups, with high accuracy observed in many prospective studies, despite the relative scarcity of data specifically addressing neonatal populations [101]. Beyond these pooled estimates, several prospective pediatric studies have directly evaluated LUS findings and diagnostic performance in children with community-acquired pneumonia.

Typical ultrasonographic findings in pediatric pneumonia include: consolidations with “shred signs” and C-lines, air/fluid bronchograms, B-lines [100,102].

A primary limitation of LUS for the diagnosis of pneumonia is that lesions must reach the pleural line to be sonographically explored. This constraint persists even though pneumonic lesions generally extend to the pleura [102]. On LUS, consolidation typically appears as a hypoechoic region compared with the surrounding lung, due to the presence of fluid in the lung, with echogenicity similar to that of the liver—a finding known as the “tissue-like sign” or “hepatization of the lung”—and characterized by irregular, poorly defined margins [4,103,104]. Consolidations are often accompanied by pleural line abnormalities such as hypoechoic or fragmented aspects [104].

C-lines represent distinctive short vertical hyperechoic artifacts in LUS, generated at the irregular tissue-air interfaces within or along the periphery of small subpleural consolidations, where early distortions of airspace architecture occur prior to full consolidation. Unlike the longer, pleural-originating B-lines, C-lines produce narrower “light-beam” reverberations that fail to reach the screen’s bottom and do not erase underlying A-lines, thereby highlighting focal pre-pneumonic changes often seen in inflammatory conditions. This feature makes them valuable for timely detection and monitoring of evolving lung pathology in point-of-care scenarios [105]. “C-lines” represent a descriptive, non-universal nomenclature used by some authors to denote early pre-consolidation changes; this terminology is not universally accepted or standardised.

The “shred sign”, also known as the fractal line, is an irregular, hyperechoic border that demarcates a lung consolidation from the adjacent aerated parenchyma. This characteristic appearance, which resembles torn paper, is due to the intense scattering of the ultrasound beam by the air within the surrounding lung [24,105,106].

Air bronchograms appear on ultrasound as hyperechoic foci within areas of consolidation and can be conceptually regarded as the pulmonary analogue of aerobilia in the abdomen. Aerobilia, defined as the presence of air within the biliary tree, manifests as bright hyperechoic foci or linear echoes within the hepatic parenchyma, often accompanied by posterior shadowing or comet-tail artifacts. Despite arising in different organs, both findings reflect the same underlying mechanism: air entrapped within a soft tissue generates a strong reflective interface, producing an echogenic signature that is readily identified sonographically. In the lungs, this pattern acquires further diagnostic value [16,29,96,107,108,109].

Inflammatory consolidations can be distinguished from atelectasis because the parenchyma remains uncompressed, and the bronchial structures typically assume an arborizing configuration, referred to as “air bronchograms” [102]. The dynamic behavior of bronchograms is also a critical discriminator: static bronchograms, which remain motionless throughout the respiratory cycle, are most often associated with obstructive or resorptive atelectasis, whereas dynamic bronchograms, characterized by visible centrifugal motion of air during breathing, are considered a reliable sign of pneumonia [103]. Additionally, the morphological appearance provides diagnostic specificity, with arborizing, tree-like branching patterns strongly suggestive of pneumonic consolidation, while parallel, non-branching linear configurations more commonly reflect the uniform compression or collapse observed in atelectasis [102,107]. This sonographic feature has also been proposed as a tool to monitor disease recovery [103]. However, the distinction is not always straightforward, as the sonographic features of atelectasis and pneumonia may overlap, limiting the ability of LUS to reliably differentiate between these conditions without clinical correlation [110].

Fluid or mucus bronchograms, visualized as echo-free tubular structures, also support pneumonia diagnosis [29]. Like fluid bronchograms, blood vessels appear as anechoic, tubular structures within the lung parenchyma; however, their walls are generally thinner and less defined than bronchial walls. The key distinguishing feature is their Doppler signal, which indicates blood flow [29,96,109]. Finally, a normal vascular branching pattern on Doppler distinguishes consolidation from lung masses [29,96].

However, it has to be noted that the sonographic appearance of lung consolidations is not disease-specific. Malignant lung lesions may present with highly variable sizes, shapes, echogenicity, and margins, and in the absence of clear invasive features, conventional B-mode findings are generally non-specific for malignancy [111]. Therefore, ultrasound findings alone cannot reliably distinguish inflammatory from neoplastic processes. The main typical findings are shown in Figure 5.

Pleural effusion will be further discussed in the paragraph “Pleural effusion”.

The ultrasonographic patterns of pneumonia often differ based on the underlying etiology. While certain etiologies may exhibit characteristic ultrasonographic signatures, significant overlap among pathogen-induced lung insults frequently necessitates integrating LUS with clinical and laboratory parameters to enhance diagnostic specificity. Bacterial infections are typically characterized by a consolidation-type pattern, where the inflammation is anatomically contained by lobar fissures or interlobular septa. In contrast, viral or Mycoplasma infections tend to show predominant interstitial involvement and can be visualized as confluent B-lines [16]. These B-lines can become so dense that they create a “white lung” appearance, reflecting an accumulation of inflammatory exudate within the interstitial spaces. In the neonatal period, mixed forms are sometimes observed, in which focal consolidations coexist with interstitial changes [4].

Several pediatric primary studies have explored whether combinations of LUS features may nevertheless contribute to etiological assessment, sometimes comparing its performance to that of clinical findings and CXR. In a prospective study of 186 children aged 1 month to 17 years hospitalized with acute lower respiratory tract infection, Buonsenso et al. [112] found significant differences among bacterial, viral, and atypical infections in consolidation size, number and distribution, air-bronchogram characteristics, pleural effusion, and the distribution of vertical artifacts. These findings suggest that LUS patterns may provide supportive etiological information when interpreted together with microbiological and clinical data, rather than functioning as stand-alone diagnostic criteria. A bunch of studies also described specific sonographic patterns for viral lower respiratory tract infections and bronchiolitis [113,114,115,116]. Furthermore, dynamic changes in LUS features such as bronchograms, consolidation size, and pleural effusion after 48 h of treatment were shown to predict antibiotic response better than clinical or laboratory parameters [112,117,118].

Berce et al. [119] prospectively evaluated 147 children with community-acquired pneumonia (CAP) and showed that consolidations in viral pneumonia were significantly smaller (median 15 mm) compared with atypical bacterial (20 mm) and bacterial pneumonias (30 mm). A 21-mm consolidation-size threshold was proposed for differentiating bacterial CAP, with a sensitivity of 80% and specificity of 75%. However, this threshold was derived from a single cohort and should be interpreted as an exploratory study-specific discriminator rather than as a clinically validated cutoff. Similarly, Malla et al. [120] proposed that subpleural consolidations > 20 mm were indicative of bacterial pneumonia, while lesions between 5 and 20 mm were more suggestive of viral disease. In their cohort of 200 children younger than 12 years with suspected pneumonia, they reported high sensitivity and specificity of LUS for bacterial pneumonia (91% and 91.3%) and somewhat lower accuracy for viral pneumonia (78.4% and 90.4%), concluding that ultrasound can reduce unnecessary antibiotic use.

Beyond consolidation size, Buonsenso et al. [113] identified additional LUS features with diagnostic value in their prospective study of 186 children aged 1 month to 17 years hospitalized with acute lower respiratory tract infection. Air bronchograms were more common in bacterial and atypical infections, with fluid bronchograms almost exclusively described in bacterial cases. Complicated pleural effusions were not observed in viral infections. Vertical artifacts also differed: in bacterial pneumonia they were mostly located adjacent to the main consolidation, while in viral and atypical forms they were diffusely distributed and often bilateral. These findings suggest that LUS patterns may provide supportive etiological information when interpreted together with microbiological and clinical data, rather than functioning as stand-alone diagnostic criteria.

Recently, Morello et al. [100] showed through a stepwise approach of models that the utility of LUS and CXR is increasingly enhanced when used in combination with clinical and laboratory data. Moreover, in addition to what has been previously reported in the literature, their study demonstrated that the performances of CXR and LUS models for the detection of pneumonia are highly overlapping [100]. Taken together, these studies indicate that LUS may contribute to etiological stratification, particularly when multiple sonographic features are considered together, but currently available thresholds and patterns remain study-specific and have not been independently validated for routine clinical decision-making. Moreover, these favourable estimates are not entirely consistent across studies and may be influenced by differences in disease prevalence, patient selection, operator training, and reference standards; moreover, the exclusion of deep or non-pleural lesions may lead to an overestimation of diagnostic performance in cohorts enriched for peripheral consolidations. Although LUS demonstrates high sensitivity in identifying pulmonary consolidations, it cannot establish a definitive etiological diagnosis of pneumonia, as sonographic patterns frequently overlap across infectious and non-infectious etiologies.

LUS has a pivotal role in the evaluation of complications as well. Pulmonary abscess, a necrotic cavitary lesion with fluid content, is a rare but serious consequence of CAP [121]. While CXR and computed tomography (CT) remain the standard diagnostic tools, LUS has demonstrated significant utility, especially in the emergency setting, providing rapid bedside recognition without ionizing radiation exposure [99,122,123]. The LUS characteristics of abscesses typically include indistinct or blurred outer margins, irregular and variably thickened walls, and a generally oval or round configuration of the lesion. At the level of the pleural interface, abscesses often form an acute chest wall angle, a finding that helps differentiate them from other pleural-based abnormalities [122,123]. Lung abscesses are frequently accompanied by peri-lesional inflammatory changes and partial collapse of adjacent lung parenchyma, creating the appearance of a thickened capsule wall [124].

An artifact that can sometimes be observed is the twinkle artifact: a Color Doppler phenomenon characterized by a rapidly fluctuating, mosaic-like signal that appears just behind highly reflective surfaces such as stones, calcifications, or trapped air. It creates a “sparkling” effect that can make small or otherwise inconspicuous structures—like gas bubbles within an abscess—easier to identify compared with standard gray-scale imaging [19,33].

An important diagnostic application of LUS is the differentiation between pulmonary abscess and empyema. On Color Doppler imaging, abscesses typically demonstrate intralesional vascular signals corresponding to residual necrotic parenchyma, whereas empyemas characteristically appear avascular [125]. Additional sonographic features proposed for differentiating pyopneumothorax from pulmonary abscess include the presence of air–fluid levels associated with the “sinusoid sign” in which these levels shift synchronously with respiration, absence of pleural sliding above the fluid level, and the “suspended microbubble sign” characterized by hyperechoic foci with trailing artifacts that move irregularly within the pleural effusion during breathing. Collectively, these findings are more consistent with pyopneumothorax [126].

From a technical standpoint, the evaluation of pulmonary abscesses is best performed with a wide-footprint transducer that offers sufficient depth penetration for assessing deep lung parenchyma. The addition of color Doppler imaging further enhances differentiation of abscesses or effusions from other entities such as consolidations, vascular malformations, or masses exhibiting internal vascularity [122,123]. Finally, LUS can be a useful guide for therapeutic interventions. It can assist in drainage procedures, which are needed in selected pediatric patients with large, persistent, complicated, or medically refractory lung abscesses [127]. An example of lung abscess is represented in Figure 6.

Beyond diagnosis, LUS may also represent a useful tool for monitoring the evolution of pulmonary consolidations over time. Although pediatric evidence remains limited, some studies have shown a progressive reduction in consolidation size during follow-up examinations [1,128]. These findings suggest that reductions in consolidation size or in the overall LUS-detected burden of pulmonary abnormalities may potentially serve as surrogate markers of treatment response [129].

### 6.2. Atelectasis

Pulmonary atelectasis is defined as the collapse of non-aerated lung tissue, caused either by parenchymal compression (non-obstructive) or bronchial blockage (obstructive) [29]. In infants, the condition most often results from airway obstruction due to mucus or meconium, leading to distal air trapping and collapse once the trapped air is absorbed. A specific form, resorptive atelectasis, is strongly linked to mechanical ventilation when delivered at insufficient pressures [37].

On LUS, pulmonary atelectasis may appear as reduced aeration with scattered or confluent B-lines, progressing to complete collapse with subpleural consolidations and static air bronchograms [130]. A key marker is the lung pulse—absence of sliding with pleural movement synchronized to cardiac activity—described by Lichtenstein with 93% sensitivity and 100% specificity for complete atelectasis [25,130].

The most distinguishing ultrasonographic finding is the presence of a static air bronchogram, which arises from air trapped within collapsed bronchi and is characterized by its lack of movement throughout the respiratory cycle. This feature contrasts markedly with the dynamic air bronchogram observed in inflammatory pulmonary consolidations, where hyperechoic foci (the air within bronchi) display synchronous motion with inspiration and expiration, thereby effectively excluding atelectasis as a diagnosis. The differentiation between these two sonographic patterns provides significant diagnostic value, as the detection of a dynamic air bronchogram is highly specific for pneumonia, while a static air bronchogram supports the diagnosis of atelectasis, reflecting non-patent bronchial pathways and isolated intrabronchial air, although it is less specific, as it may also occur in pneumonia [107,131,132,133,134,135].

Other supportive signs include pleural line irregularities, absent lung sliding in obstructive atelectasis, or persistent sliding in early stages of compression atelectasis (e.g., from effusion) until air is fully resorbed [17,136].

In cases of near-complete hemithorax opacification, LUS is highly sensitive for distinguishing atelectasis or consolidation from pleural effusion—an assessment often not possible with CXR [29,137]. LUS has also shown excellent accuracy for pediatric atelectasis, with studies reporting a sensitivity of 100% [107]. An example of atelectasis is shown in Figure 7.

Furthermore, LUS enables real-time, non-invasive monitoring of dynamic changes in lung aeration during recruitment maneuvers, offering a practical bedside tool to guide respiratory management in mechanically ventilated children. In the study by Tusman et al., the authors investigated the utility of LUS to evaluate postural lung recruitment in a cohort of mechanically ventilated pediatric patients [138]. The study employed a standardized protocol in which children were moved from supine to lateral decubitus positions, and LUS was performed before and after positional changes to assess regional aeration in dependent and non-dependent lung zones. The authors reported that postural recruitment resulted in significant improvements in aeration scores in non-dependent lung regions, with a concomitant reduction in LUS signs of atelectasis and consolidation. These LUS-detected changes were associated with improvements in oxygenation parameters, suggesting that LUS findings reflected physiologically meaningful recruitment. Noteworthy, the study demonstrated excellent interobserver agreement for LUS aeration scoring, supporting the reproducibility of LUS-based assessment in the pediatric intensive care setting. These findings indicate that LUS can serve not only as a diagnostic modality but also as a monitoring tool to titrate recruitment strategies, optimize ventilator settings, and potentially minimize the risk of ventilator-induced lung injury by avoiding excessive or insufficient recruitment.

### 6.3. Bronchiolitis

Bronchiolitis represents one of the most common reasons for pediatric emergency visits [139]. Its diagnosis is primarily clinical; imaging is not recommended by official guidelines but is occasionally employed in practice [140]. This viral lower respiratory tract infection typically affects children under two years, with the most severe forms seen in infants younger than three months [16,104,141]. The disease is driven by airway edema, mucus hypersecretion, and necrosis of bronchiolar epithelium, leading to obstruction of distal airways and contributing to its classification as a frequent pediatric cause of alveolar-interstitial syndrome (AIS) [16,104,141].

LUS has recently proven valuable in evaluating and managing bronchiolitis [115,142,143,144]. Characteristic findings include small subpleural consolidations (<1–1.5 cm, hypoechoic with irregular borders), pleural line irregularities, and especially B-lines, which may appear scattered in mild disease, become coalescent or confluent in more severe forms, progressing up to a “white lung” pattern [104,140,145,146]. Noteworthy, the extent of ultrasound abnormalities correlates with clinical severity, making LUS a useful bedside tool not only for diagnosis but also for prognostic assessment [140,145].

Several studies have shown that LUS findings and scoring systems correlate with disease severity, hospitalization length, oxygen requirement, and even Pediatric Intensive Care Unit (PICU) admission in infants with bronchiolitis [147,148,149].

Even if LUS findings in bronchiolitis can be valuable for management, they are not pathognomonic for the condition [16,142,145,150,151,152,153]. The aforementioned typical features reflect interstitial inflammation and regional hypoventilation. In some cases, inflammation extends to the alveoli, producing larger consolidations with dynamic air bronchograms [142,150]. The most frequently and significantly affected lung fields are the posterior and inferior regions. Basile and colleagues proposed an ultrasound classification linked to clinical severity, showing good agreement between imaging and symptoms. In particular, subpleural consolidations > 10 mm were associated with a worse clinical course [16,142,150,152]. Moreover, even very small subpleural consolidations (<0.5–1 cm), often accompanied by adjacent B-lines, have been linked to areas of impaired ventilation in viral pneumonia and bronchiolitis [16,109,154].

In recent years, LUS has been increasingly used in the evaluation of bronchiolitis, particularly in young children, where its non-invasive, radiation-free, and bedside nature makes it ideal for both diagnosis and follow-up [16,153,155]. Compared with CXR, LUS is more sensitive in detecting small subpleural consolidations (<2 cm) [153] and can also identify bacterial superinfection through the presence of larger consolidations with air bronchograms [16,113]. Overall, LUS is considered more reliable than CXR in bronchiolitis and correlates closely with clinical progression [104,145].

Biagi et al. reported a sensitivity of 100% and specificity of 83.9% for detecting pneumonia in infants with bronchiolitis, compared with 96% and 87.1% for CXR [113,140]. Basile et al. found that LUS could identify infants requiring supplemental oxygen with 97% sensitivity and 99% specificity [150].

A LUS scoring system has been developed, with higher scores assigned based on the presence of confluent B-lines, widespread B-lines, and larger consolidations. This score has been shown to correlate with key clinical outcomes, including disease severity, duration of hospitalization, and the need for oxygen therapy [144,146]. However, evidence supporting the prognostic value of LUS scores in bronchiolitis remains preliminary, as most studies are observational, use non-uniform scoring systems and clinical endpoints, and may be affected by verification and incorporation bias because LUS findings are often interpreted alongside the same clinical variables used to define disease severity.

An example of bronchiolitis is shown in Figure 8.

### 6.4. Pleural Effusion

LUS ability to detect pleural effusion is widely confirmed in the literature [16,96,108,156], with a sensitivity and specificity close to 100% [140].

Pleuropneumonia is the condition in which pleural effusion is most frequently detected in the pediatric population. Other associated conditions include: heart failure, kidney disease, liver cirrhosis, pancreatitis, fluid overload, trauma, chylothorax, and paraneoplastic effusion [157].

Much of the quantitative diagnostic-performance evidence for pleural effusion detection derives from adult or mixed populations. In these studies, when compared with CXR, LUS has been shown to exhibit greater sensitivity and accuracy in the detection of pleural effusion. LUS has been demonstrated to detect effusions as small as 10–20 mL, with a sensitivity of 100% for effusions >100 mL [140]. Conversely, CXR has been demonstrated to detect the presence of pleural effusion in patients in an orthostatic position, provided that the volume of the effusion is at least 150–200 mL. It should be noted that the sensitivity of this method decreases in the supine position [16,37,96,108,158,159,160,161]. CXR has overall moderate sensitivity and reasonably high specificity [157,162,163,164].

Current global pulmonary guidelines recommend LUS as a key component of the initial imaging evaluation for the diagnosis and assessment of pleural effusion [157,162,165,166].

When performing LUS assessment of pleural effusion, it is correct to start at the level of the diaphragm. The key anatomical landmarks that should be recognized are: the liver or spleen, the diaphragm, the chest wall, the lung, and the effusion itself. In sonography, pleural effusion typically appears as an anechoic space above the diaphragm, accompanied by loss of the normal mirror image of the liver or spleen within the lung fields. In cases of large effusion, compression of the adjacent lung may produce echotexture similar to hepatic parenchyma [140].

In pediatric patients, pleural effusion usually accumulates in the inferior thoracic regions, and the liver and spleen provide practical acoustic windows for evaluating the supradiaphragmatic pleural cavities and lung bases. Whenever possible, examination from the dorsal side in a sitting or standing child improves both visualization and quantification of fluid, since free effusions collect in the lowest spaces, whereas in the supine position they localize posteriorly [16,157]. The posterior axillary line is considered the optimal intercostal site for longitudinal scans, but the entire thoracic cavity should be assessed because effusions may be loculated [157]. For best image quality, a high-frequency linear probe is recommended, while in extensive effusions a convex transducer may be required to ensure adequate penetration [157].

LUS allows precise assessment of pleural fluid volume and selection of the safest drainage site. The 97% procedural success estimate derives from adult studies; pediatric practice supports ultrasound guidance, but direct pediatric comparative data are more limited [104,167].

Ultrasound is also a well-established tool for identifying the optimal site for diagnostic or therapeutic aspiration [41,168]. When reporting site marking for drainage, it is important to specify whether the scan was performed with the patient upright or supine [96]. Current guidelines recommend ultrasound guidance during thoracentesis, as it improves procedural success and reduces complications-the most frequent being PTX-although operator expertise remains a critical factor [140,169,170]. Evidence from predominantly adult and mixed-population studies indicates that pleural fluid may show different sonographic appearances. Pleural effusions show different echogenic patterns on ultrasound depending on their nature [16,38,96,108,156,158,159]. Simple pleural effusions often appear as anechoic fluid collections between the pleural layers and may change with patient position. Respiratory or cardiac motion of the adjacent lung may generate dynamic signs such as the “sinusoid” sign and the “pulse” sign. However, sonographic appearance alone cannot reliably distinguish transudative from exudative pleural effusions. In patients with suspected heart failure, the coexistence of bilateral diffuse B-lines and pleural effusion may support a cardiogenic congestive pattern [16,171], but this finding should be interpreted in the overall clinical and hemodynamic context rather than as proof of a transudative effusion.

M-mode can help visualize the “sinusoid sign”, a sinusoidal movement of the collapsed but expandable lung in the fluid, synchronized with respiratory acts [168].

Additionally, LUS provides valuable qualitative information on pleural fluid characteristics. Exudates often demonstrate internal echoes, reflecting protein, cells, or blood, as well as fibrin strands or septations. Their morphology differs according to etiology and duration of the condition. Echogenic or septated effusions, together with pleural thickening or adjacent parenchymal changes, strongly suggest an exudative nature [16,140,172].

When rich in protein, blood, or cells, exudates may contain hyperechoic floating particles that move with ventilation [173]. The “fluid color” sign is a diagnostic ultrasound feature observed with color Doppler, useful in differentiating pleural effusion from pleural thickening or solid tissue. When color Doppler is applied, true pleural fluid will show a color signal within the collection in synchrony with respiratory or cardiac movement—this indicates the presence of mobile fluid rather than stationary tissue. In contrast, pleural stationary thickening or solid masses do not produce such a color signal because they lack internal movement. The presence of the fluid color sign is highly sensitive and specific for detecting even minimal or loculated pleural effusions and is especially valuable when traditional grayscale imaging cannot clearly distinguish between effusion and thickening [5].

Homogeneous hyperechoic effusions are most often seen in empyema or hemothorax, and additional findings such as pleural thickening, nodularity, or adjacent consolidations with dynamic air bronchograms favor an infectious exudate [16,38,96,108,156,158,159].

The complexity of exudates can range from low-grade (mild floating particles, e.g., infection, hemorrhage) to high-grade (dense echogenicity, septations, fibrin, pleural thickening), with the latter typically representing organized infection, empyema, long-standing hemothorax, or malignant effusions [37,96,174].

Specific examples include hemothorax, which varies depending on stage, with chronic cases showing thick septa [173]; and empyema, usually encapsulated and not free-expanding, homogeneous, and associated with pleural thickening and adjacent atelectasis, though this classic picture is present in about only 40% of cases [175].

Such complicated effusions, encapsulated or septated, are more prone to adverse outcomes, may complicate thoracentesis, and can favor infections like tuberculosis [96]. Overall, these features help identify the nature of the effusion and may support clinical decision-making, although they require integration with clinical and laboratory data.

The mirror effect occurs when echoes undergo multiple reflections at a strongly reflective interface, creating a duplicated image positioned deeper than the true structure [176]. In LUS, this is typically seen at the diaphragm, where the pleural-air interface acts as the mirror, making the liver or spleen appear within the thoracic cavity. A mirror artifact in this region is a normal finding and suggests absence of pleural effusion. When fluid is present, it appears as an anechoic triangular space above the diaphragm and allows vertebral bodies or proximal ribs to appear into the thorax (“spine sign”). With larger effusions, consolidated or atelectatic lung tissue may also be seen moving within the anechoic collection, creating the “jellyfish sign” [66,176].

In LUS, pleural effusion appears confined between nearly parallel pleural layers, and together with adjacent rib shadows, these boundaries form four sides of a quadrilateral shape known as the “quad sign” [131].

Two examples of pleural effusions are represented in Figure 9.

Several ultrasound techniques have been proposed to quantify pleural effusion, but all rely on multiple measurements, and many authors argue that exact volume calculation has limited clinical relevance [16]. A qualitative approach may therefore be more practical, as LUS not only identifies effusion but also demonstrates its impact on the lung parenchyma by showing different degrees of parenchymal collapse and thus atelectasis [16,168].

The utility of quantification for follow-up and assessing therapeutic response is recognized; however, the accuracy and feasibility of different formulas vary. Therefore, it is essential to ensure consistency in the technique between baseline and follow-up measurements to minimize bias. The most reliable estimations are obtained in sitting patients with free-flowing effusions, though pediatric evidence is still limited [157].

Most commonly used sonographic formulas for pleural effusion volume estimation were developed and validated primarily in adult critical-care populations, and their applicability to children remains uncertain. Among these adult-derived methods, commonly used formulas include Goecke’s equations: (1) craniocaudal extent (cm) of the effusion at the dorsolateral chest wall measured in erect position with the probe oriented longitudinally (x) × 90 = effusion volume (mL); (2) (basal lung-mid-diaphragm distance + x) × 70 = effusion volume (mL) [177]. Another adult-derived method, Balik’s formula, estimates effusion volume-especially in critically ill patients in supine positions with the probe oriented transversally by multiplying the pleural effusion thickness (mm) at the posterior axillary line by a factor of 20 [178]. However, in supine patients, accuracy decreases since effusions mainly collect posteriorly due to gravity [66,179], making small or moderate effusions less accessible to standard scanning windows and more difficult to visualize. Another option is the conventional ellipsoid method, multiplying length × width × height (cm) × 0.52, which is widely applied to other organs as well [180].

Although a variety of alternative methods are available, the most important factor for achieving reliable and comparable results among sonographers is the implementation of a standardized approach within each department [157]. Although several ultrasound formulas have been proposed to estimate pleural effusion volume, the best-known methods, including the ellipsoid method and those of Balik and Goecke, were developed and validated mainly in adult critical care populations, as previously stated. Their applicability to children remains uncertain because of anatomical and physiological differences, as well as the smaller thoracic dimensions and different fluid distribution patterns in pediatric patients. Therefore, extrapolation of these adult-derived formulas to children should be approached with caution, and a qualitative or longitudinally standardized assessment is generally preferable in pediatric practice.

### 6.5. Pulmonary Edema

LUS is a reliable tool for diagnosing pulmonary edema, a condition resulting from fluid accumulation in the alveoli and interstitial spaces due to both cardiogenic and non-cardiogenic mechanisms [140]. The presence of edema leads to a reduction in lung aeration [130]. Much of the evidence supporting the diagnostic performance of LUS for pulmonary edema derives from adult populations, particularly patients with acute dyspnea or acute decompensated heart failure. In these settings, compared with conventional CXR, LUS offers greater sensitivity for pulmonary edema, a difference that has the potential to enhance patient management and prognosis [14,17,181,182,183,184]. Pediatric-specific validation remains more limited.

In pulmonary edema, LUS commonly shows an increased number and density of B-lines. These artifacts are associated with altered peripheral lung aeration and may correlate with increased extravascular lung water [140], but they should not be interpreted as direct visualization of interstitial septal thickening or alveolar flooding.

Adult critical-care and heart-failure literature has described several B-line patterns associated with pulmonary edema. In these studies, the key finding is the presence of three or more B-lines in a single interspace or a confluent line occupying the interspace [184]. The number and distribution of B-lines vary depending on the extent of loss of lung aeration and interstitial/alveolar edema, changes also seen as ground-glass opacities on CT scan [17,185,186,187].

The pattern of B-lines may provide information on the extent of lung aeration loss: well-separated lines are generally associated with milder interstitial edema, while confluent or “waterfall” B-lines are more often observed in more extensive alveolar involvement [104,130,188]. Earlier adult-derived descriptions proposed that B-lines spaced 7 mm apart correspond to thickened interlobular septa and interstitial edema, whereas those <3 mm apart are linked to alveolar edema with ground-glass changes [188]. However, these distance-based patterns should be regarded as descriptive associations rather than direct anatomical surrogates, as B-line morphology is also influenced by technical and acquisition factors. Distribution also matters—focal B-lines may occur in localized disease such as pneumonia, atelectasis, or neoplasm, whereas a diffuse bilateral pattern may be seen in cardiogenic pulmonary edema, acute respiratory distress syndrome (ARDS), lung contusion or pulmonary fibrosis [104,189,190]. Finally, a gradual reduction in the number and density of B-lines—from confluent to separated, and eventually their disappearance—may parallel decreased extravascular lung water and effective response to therapy [140,191]. In pulmonary edema, lung sliding is typically maintained [17,186,190]. According to international literature, diagnosis relies on seven sonographic features of B-lines: (1) comet tail, (2) origin from the pleural line, (3) movement with lung sliding, (4) vertical extension, (5) well-defined borders, (6) hyperechogenicity, and (7) obliteration of A-lines [130,131]. Adult studies have suggested that certain combinations of pleural and B-line findings may help distinguish cardiogenic pulmonary edema from noncardiogenic alveolar-interstitial syndrome (AIS). The presence of a fragmented pleural line, vertical subpleural patterns, spared areas of normal lung parenchyma, and reduced pleural motion are indicative of non-cardiogenic causes. In contrast, an uninterrupted pleural line with a homogeneous distribution of B-lines suggests cardiogenic edema [140,192]. In general, AIS can be characterized by pleural line abnormalities, multiple B-lines, subpleural consolidations, and possible loss of sliding due to exudate adherence [104,193]. However, much of the evidence supporting the diagnostic performance of LUS in pulmonary edema derives from adult populations, particularly patients with acute dyspnea or acute decompensated heart failure, whereas pediatric-specific validation remains more limited.

In adult critical-care practice, LUS has also been incorporated into fluid-management algorithms, since the appearance of B-lines may indicate pulmonary edema, as described in the FALLS protocol [28,194]. Yet, evidence directly linking B-lines to fluid-induced respiratory deterioration is lacking. Moreover, bilateral B-lines are nonspecific: they may also occur in viral pneumonia (e.g., COVID-19), ARDS, and interstitial lung disease, whereas in cardiogenic pulmonary edema they often coexist with bilateral effusions [17,195,196]. An example of pulmonary edema is represented in Figure 10.

In recent years, a multimodal ultrasound approach combining LUS with hemodynamic assessment has been proposed in adult hemodynamic ultrasound literature to improve clinical decision-making in acute settings. In particular, evaluation of the inferior vena cava (IVC) diameter and its respiratory variation may provide additional information on intravascular volume status [197]. A dilated IVC with reduced collapsibility is generally associated with elevated right atrial pressure, whereas a small and highly collapsible IVC may suggest hypovolemia [194]. However, the role of IVC measurements in pediatric patients remains incompletely defined, and parameters such as the collapsibility index or the IVC/aorta ratio require age-specific validation. Therefore, these parameters should be interpreted with caution and always integrated with clinical context. The combined use of lung, cardiac, and vascular ultrasound may offer a more comprehensive assessment of the underlying pathophysiology, particularly in differentiating cardiogenic from non-cardiogenic causes of respiratory distress and guiding fluid management. While this approach has shown promising results in adult populations, pediatric-specific evidence is still limited and warrants further investigation. Accordingly, the diagnostic and management implications of B-line patterns in children remain uncertain, since pediatric studies are relatively sparse, thresholds are not uniformly defined, and B-lines may reflect several distinct pathophysiological processes rather than pulmonary edema specifically.

The application of LUS to pulmonary edema should also be considered in the context of congenital heart disease (CHD) with pulmonary overcirculation, where increased pulmonary blood flow leads to interstitial and alveolar fluid accumulation analogous to cardiogenic pulmonary edema [198].

In this setting, LUS has been specifically investigated as a bedside tool to detect and grade pulmonary overflow in neonates and children with CHD, showing high sensitivity and specificity compared with CXR and, in some studies, CT [198].

The LUSNEHDI (Lung Ultrasound in Neonatal Congenital Heart Disease) study by Rodríguez-Fanjul et al. demonstrated that neonates with CHD predisposed to pulmonary overflow developed a higher B-line score after 72 h of life, with good correlation to echocardiographic findings and superior performance to physical examination and CXR.

In a subsequent prospective study of newborns with critical CHD and increased pulmonary blood flow [199], Kaya et al. reported that LUS-detected pulmonary edema increased on the first postoperative day compared with baseline and then progressively decreased by day 6 after surgery, mirroring the expected clinical trajectory. The LUS findings exhibited trends parallel to those of serum pro-BNP levels, need for respiratory support, and diuretic requirements [199]. These findings support the use of LUS as a dynamic, bedside tool to monitor pulmonary congestion in this population with CHD, with potential implications for guiding diuretic therapy and integrating imaging findings with clinical status and biomarkers such as natriuretic peptides.

### 6.6. Pneumothorax

It has been widely proven that LUS can detect PTX with much higher sensitivity, specificity and accuracy than CXR [96,185,200,201], although much of the available performance evidence derives from adult emergency and critical-care cohorts. Dedicated pediatric and neonatal studies support its diagnostic utility, but the pediatric evidence base remains smaller so far. As a fast point-of-care technique, LUS enables quick and focused management of critical scenarios, particularly in trauma cases [17]. Because air naturally rises to the highest parts of the chest, the best position for LUS to detect a PTX is supine, which lets air gather in the anterior chest wall at the 2nd–4th intercostal spaces along the midclavicular lines [17,185]. If the patient is not lying supine or flat, a scan of the lateral chest can be evaluated to rule out PTX [104].

In PTX, air gathers between the two pleural sheets, resulting in collapse of the underlying lung parenchyma [140]. This trapped air reflects the ultrasound beam. Additionally, the visceral pleura becomes invisible and fails to slide against the parietal pleura [14,29,134,202].

This leads to a series of LUS signs, including absence of pleural sliding, presence of lung point, visualization of A-lines due to ultrasound reflection from intrapleural air, absence of B-lines and lung pulse and, in M-mode, the “barcode sign” or “stratosphere sign”.

As with adults, the sonographic hallmark of PTX is the absence of pleural sliding, along with the possible detection of the “lung point” in B-mode. This finding has 79% sensitivity and 100% specificity for diagnosing PTX [96,203]. It represents the ultrasound visualization of the transition zone between the margin of the PTX, where the pleural layers are separated, and the normally aerated lung with intact pleural apposition [4]. Nevertheless, a complete PTX will not show this transition [17]. It is also important to understand that other lung conditions, such as pulmonary adhesions, atelectasis, and apnea, are associated with the absence of lung sliding [17,186,202].

If the PTX is limited, a “double lung point” can be observed [204]. It consists of two lung points between which sliding and B-lines are absent. On their lateral sides, however, sliding is observed [16]. PTX evolution can be steadily monitored while maintaining radioprotection through daily US examination of lung points, which can be easily and quickly performed at the patient’s bedside [140].

Adult studies have proposed that the anatomical position of the lung point may provide an approximate indication of PTX extent. If a lung point is identified in the anterior chest, the air volume is relatively minor and likely not visible on a CXR. A lateral lung point corresponds to a more extensive PTX, while a posterior or absent lung point corresponds to a massive PTX [130,205]. In M-mode, PTX produces the “barcode sign” or “stratosphere sign,” which is characterized by a series of parallel horizontal lines. The lung point is identified on ultrasound as the junction where a segment displaying the “barcode sign” (a linear pattern consistent with absent lung sliding) abuts a segment exhibiting the physiological “seashore sign”, with a sand-like pattern below the “waves” of the pleural line, corresponding to pleural sliding [4,16,27,140]. The air between the pleural layers causes the chest wall and lung to be still, resulting in the “barcode sign” [16]. Both M-mode signs are shown in Figure 11.

PTX may be associated with subcutaneous emphysema, especially in traumatic scenarios. In this case, it is important to understand that lung sliding will not be visible because air collected in the subcutaneous tissue blocks ultrasound beams [21]. In subcutaneous emphysema, vertical hyperechoic artifacts called E-lines may appear and be mistaken for B-lines; however, unlike B-lines, they do not originate at the pleural line and show no respiratory movement [21,173]. Subcutaneous emphysema will also impede visualization of the “bat sign” [17].

Importantly, pediatric-specific primary evidence also supports the diagnostic role of LUS for PTX. In a prospective observational study of children presenting to the emergency department with acute chest pain, Scialanga et al. directly evaluated LUS for suspected PTX, confirming the diagnostic value of specific sonographic signs in the non-neonatal pediatric population [206]. In critically ill neonates, Raimondi et al. prospectively demonstrated that LUS can provide rapid and accurate bedside diagnosis of PTX, extending the evidence to the neonatal intensive-care setting [207]. Nevertheless, it should be noted that much of the evidence regarding LUS accuracy for PTX derives from adult emergencies and critical care populations; dedicated pediatric validation remains more limited.

Moreover, the diagnostic performance of LUS in PTX remains subject to important limitations and ongoing debate. Although several studies have reported high sensitivity, more recent evidence suggests that its accuracy may be lower than previously described, while still remaining superior to CXR in trauma and critical care settings [208]. As previously stated, LUS only allows partial visualization of the pleural surface, as some areas remain inaccessible due to rib shadowing and mediastinal structures. In addition, interpretation of sonographic findings may be challenging, as several conditions—such as lung bullae, contusions, pleural adhesions, or hyperinflation—can produce false-positive results or mimic PTX [14,209]. Importantly, the absence of lung sliding alone is not specific for PTX and may also be observed in other clinical conditions, including atelectasis, consolidation, or lung contusion [209]. In particular, differentiating PTX from bullous lung disease remains particularly challenging, as both conditions may produce overlapping sonographic findings. In these cases, ultrasound signs alone may not reliably distinguish between entities, and integration with clinical context and, when necessary, confirmatory imaging such as CT scan is essential to avoid misdiagnosis [210].

The absence of lung sliding, although commonly considered a key sign, is not specific and may also be observed in other conditions associated with reduced pleural motion, including severe asthma, where pleural line abnormalities and reduced or absent gliding have been described [211].

Patient-related factors may further reduce diagnostic accuracy. In particular, morbid obesity may impair image resolution and has been associated with false-positive findings, including apparent absence of lung sliding due to acoustic artefacts related to altered ultrasound propagation in adipose tissue [212,213].

Therefore, diagnosis often requires a combined interpretation of multiple sonographic signs rather than reliance on a single finding. Moreover, although LUS is a reliable alternative to CT for estimating PTX extent in certain scenarios, CT imaging remains the reference standard for identifying occult cases and accurately assessing lesion volume [209]. Finally, although LUS represents a valuable bedside tool, its role in certain critical care scenarios remains debated. While it may provide rapid assessment, some evidence suggests that portable chest radiography still represents an appropriate first-line imaging modality in clinically unstable patients, including those with suspected PTX, with ultrasound serving as a complementary technique in selected cases [214].

Another relevant condition is hydropneumothorax, which is rarely reported in children, and evidence on its sonographic diagnosis is therefore limited. LUS may demonstrate the coexistence of pleural air and fluid, with loss of lung sliding and a dynamic air–fluid interface; more recently, the “air–fluid point”, representing the dynamic sonographic transition between pleural air and pleural fluid, has been described as a specific ultrasound sign supporting the diagnosis of hydropneumothorax in neonates [215]. Pediatric case reports also suggest that LUS may facilitate rapid bedside recognition and management of hydropneumothorax [216].

### 6.7. Pulmonary Contusions

Diagnosing pulmonary contusion is challenging. Conventional CXR frequently underestimates its occurrence, and reliable detection usually requires advanced imaging such as CT [217,218,219]. Most evidence on the diagnostic performance of LUS for pulmonary contusion derives from adult trauma cohorts. In these studies, evidence indicates that plain CXR identifies only severe trauma, whereas LUS can detect contusions earlier and with substantially greater sensitivity and specificity [17,186,219,220]. However, it is to be noted that, while much of the available diagnostic-performance evidence derives from adult trauma populations, dedicated pediatric data remain limited.

Adult trauma studies have described three sequential pathological/sonographic phases in lung contusions [219,221,222,223]: the initial traumatic impact causing hemorrhagic or lacerated foci in the parenchyma, an edematous stage with interstitial infiltration within 1–2 h, and subsequent alveolar flooding with blood, inflammatory cells, and debris, producing peak consolidation at 24–48 h [224]. This evolution can be further aggravated by secondary surfactant dysfunction [225,226].

LUS is an adequate tool for visualizing interstitial edema [131,184]. Thus, it can detect pulmonary contusions earlier than CXR in an emergency context [219]. Lung contusion primarily appears in LUS with B-lines, and can be associated with consolidations and C-lines as well [17,104,186,219,220,226]. Pulmonary contusion may show a focal or regionally predominant distribution of B-lines and peripheral consolidations corresponding to areas of traumatic injury on CT [219]. However, this pattern is not specific: contusions may be multifocal or bilateral, particularly after high-energy blunt trauma, and their distribution may overlap with other causes of alveolar–interstitial involvement [217]. Likewise, ARDS should not be considered uniformly diffuse, as lung involvement may be markedly heterogeneous [227], with alternating abnormal and relatively spared regions. Therefore, the distribution of B-lines or consolidations alone cannot reliably distinguish pulmonary contusion from ARDS or pulmonary edema, particularly in critically ill patients in whom mixed pathophysiological processes may coexist. In contrast, other conditions exhibit a generalized distribution. An example of lung contusion is represented in Figure 12.

If there is also a component of traumatic PTX, contusion B-lines may appear in an asymmetrical or focal pattern, laterally or dorsally near the PTX [228].

### 6.8. Tuberculosis

Pediatric pulmonary tuberculosis (PTB) remains diagnostically challenging since clinical manifestations are often nonspecific and microbiological confirmation is frequently difficult, making diagnosis dependent on the integration of clinical, epidemiological, microbiological, and imaging findings [229,230,231,232]. Chest radiography is generally the first-line imaging examination, although its sensitivity for mediastinal and hilar lymphadenopathy and its specificity for parenchymal and pleural abnormalities are limited; CT provides greater diagnostic detail but entails higher radiation exposure and is less accessible in resource-limited settings [233,234,235]. These limitations have stimulated interest in radiation-free, bedside imaging alternatives such as LUS. In this context, LUS has emerged as a potentially useful complementary imaging modality in the assessment of pediatric PTB, with applications spanning the detection of pulmonary and extrapulmonary abnormalities, follow-up, and procedural guidance.

Of the pulmonary abnormalities described by LUS in PTB, consolidations and subpleural nodules are some of the most frequently reported findings. In adults, Agostinis et al. described multiple subpleural nodules as the most frequent sonographic abnormality in a cohort of 60 patients with PTB, together with lung consolidations and other peripheral parenchymal abnormalities [236]. Similar parenchymal patterns, including consolidations and subpleural abnormalities, have also been reported across the broader literature on LUS in TB [235,237]. Pediatric studies have likewise identified consolidations and subpleural nodules as common sonographic abnormalities. In a pediatric cohort from Guinea-Bissau, consolidation and subpleural nodules were detected in 57% and 55% of children with PTB, respectively [238]. Small subpleural consolidations have been described sonographically as hypoechoic or tissue-like areas < 1 cm, with or without air bronchograms [239,240,241]. In the study by Heuvelings et al., consolidations were predefined as <0.5 cm or ≥0.5 cm for reporting purposes. Consolidations ≥ 0.5 cm were detected at a similar frequency by LUS and CXR, whereas lesions < 0.5 cm were more frequently identified by LUS. In children with suspected PTB, consolidations ≥ 0.5 cm were detected nearly as frequently by LUS as by CXR, whereas consolidations < 0.5 cm were predominantly identified by LUS [242]. The same pediatric investigations also suggested differences from adult disease, including less frequent resolution and a smaller proportional reduction in consolidation size at one-month follow-up [240]. In another prospective adult cohort, LUS sensitivity was highest for nodules (95%) and micronodules (82%), followed by consolidations with air bronchograms (72%), whereas cavitations were detected less frequently (33%) [243]. Nevertheless, particularly in children, the clinical relevance and natural history of small subpleural consolidations remain incompletely defined, and their severity and distribution in isolated pulmonary TB require further investigation [239,240,241].

Pleural involvement is a peculiar finding in pediatric PTB and represents one of the most suitable manifestations of PTB for ultrasound assessment. In the first systematic description of LUS findings in children with suspected PTB, Heuvelings et al. found pleural effusion to be more frequent in children than in previously reported adult series [240]. In another pediatric cohort, pleural effusion was among the most common LUS abnormalities, and most ultrasound abnormalities resolved within three months of treatment [229]. Compared with conventional radiography, LUS appears particularly advantageous for pleural disease: in children with suspected PTB, pleural effusion was detected significantly more often by LUS than by CXR, particularly among microbiologically confirmed cases [242]. This observation is consistent with broader pediatric and adult imaging literature showing the superiority of LUS over CXR for detecting pleural fluid and its ability to distinguish pleural effusion from adjacent pulmonary consolidation [128,242]. Beyond simple detection, LUS can characterize pleural fluid, including its volume and internal organization, and has been reported to identify features of organized pleural effusion in patients with confirmed PTB [237,240,241]. This makes LUS potentially useful not only at diagnosis but also during follow-up, including assessment of residual pleural thickening and response to local treatment [237,241,243,244]. Furthermore, once a pleural effusion has been identified, LUS can facilitate ultrasound-guided aspiration, drainage, or biopsy and may improve the safety and effectiveness of thoracocentesis performed for diagnostic or therapeutic purposes [241,245,246,247].

Mediastinal lymphadenopathy is another relevant target for LUS in pediatric PTB, especially in infants and young children, in whom the thymus provides a favorable acoustic window. Pathological lymph nodes are typically round or oval, relatively hypoechoic, and may lack a fatty hilum; internal calcification or a central anechoic/hypoechoic area reflecting necrosis may also be observed [241,245,248]. The most commonly reported locations include upper paratracheal, hilar, paratracheal and aortopulmonary-window stations, with accessibility varying according to nodal position and sonographic window [241,244,249]. Recent pediatric data confirm that LUS can reliably identify accessible mediastinal lymphadenopathy, particularly enlarged aortopulmonary-window nodes, and previous studies have shown that children with confirmed PTB may have larger mediastinal nodes than those with other respiratory diseases [242,247,250]. This may be clinically relevant because lymphadenopathy can be difficult to identify reliably on CXR, whereas LUS may reveal adenopathy even when CXR findings are normal or inconclusive [241,242,244,249]. However, sonographic performance is strongly dependent on nodal location and technique, with lower detection rates for subcarinal, right hilar and other deep nodes; moreover, lymphadenopathy can also be identified in children without PTB, so size and morphologic criteria still require further validation to improve diagnostic specificity [241,242,244].

In PTB, a diffuse distribution of B-lines and subpleural granularity across multiple lung regions has been described as a sonographic pattern suggestive of miliary involvement [241,251]. Compared with more focal parenchymal abnormalities, this pattern relies on the widespread distribution of peripheral sonographic changes rather than on a single specific lesion. However, the available evidence is limited, and these findings should be regarded as suggestive rather than pathognomonic for miliary TB, particularly because similar vertical artifacts and pleural abnormalities may occur in other diffuse pulmonary processes. Accordingly, LUS may contribute to the recognition of a miliary pattern when interpreted in the appropriate clinical and radiological context, but further pediatric studies are needed to define its diagnostic performance and specificity [241,251].

Beyond consolidations and subpleural nodules, LUS may also identify less common parenchymal and structural abnormalities in PTB when these lesions extend sufficiently close to the pleural surface. Cavitations are detectable only when peripherally located, which likely explains their lower reported sensitivity compared with nodules, micronodules, and consolidations with air bronchograms [243]. This reflects a fundamental limitation of LUS, as lesions that remain deep within aerated lung are not accessible to the ultrasound beam.

Overall, the diagnostic value of LUS in PTB is limited by the fact that most sonographic abnormalities are not disease-specific. In a recent prospective pediatric study by Munyangaju et al. [250], LUS showed modest sensitivity but relatively high specificity for selected TB-related abnormalities when assessed against a composite clinical, microbiological, and imaging reference standard; in particular, accessible findings such as enlarged aortopulmonary-window lymph nodes and small subpleural consolidations appeared relatively specific for TB in the appropriate clinical context [250]. However, this specificity cannot be generalized to all LUS findings. Subpleural nodules, for example, are also frequently observed in other lower respiratory tract infections, including COVID-19, bacterial pneumonia, and Pneumocystis pneumonia [247,252]. Similar uncertainty applies to mediastinal lymphadenopathy in children: enlarged nodes may also be detected in patients considered unlikely to have PTB, making differentiation from other respiratory infections difficult and highlighting the need for validated size and morphologic criteria [242]. Likewise, the clinical significance of small subpleural consolidations remains incompletely defined. All in all, these observations suggest that individual LUS abnormalities should not be interpreted as pathognomonic for PTB, but rather as components of a broader diagnostic assessment integrating sonographic patterns with clinical, epidemiological, microbiological, and other imaging findings [247,250].

In general, current evidence supports LUS as a complementary rather than replacement imaging tool in PTB, with potential roles in triage, detection of accessible parenchymal and pleural abnormalities, assessment of mediastinal lymphadenopathy, treatment follow-up, and guidance of pleural or pericardial procedures [237,241,244,247]. However, important limitations remain: aerated lung restricts visualization to pleural and subpleural lesions; thus, deep mediastinal, hilar, retrotracheal, and subcarinal abnormalities may be missed, operator experience and acquisition technique affect performance, and several findings are not specific for PTB [242,247,248,250]. Accordingly, CT remains superior for detailed assessment of deep thoracic structures, although its use in children must be balanced against radiation exposure, whereas MRI represents a radiation-free alternative where infrastructure and expertise are available [233,253,254,255]. The evidence base, especially in children, remains limited and heterogeneous, with no consensus on standardized acquisition protocols, diagnostic thresholds, or the precise role of LUS as a screening test [235,252,256].

A thorough list of the artifacts and signs analyzed in this text is provided in Table 3.

## 7. Neonatal Applications of LUS

In neonates, LUS is particularly useful for the bedside evaluation and monitoring of several common causes of early respiratory distress [54]. In TTN (transient tachypnea of the newborn), LUS typically demonstrates bilateral interstitial syndrome with multiple B-lines, often showing a characteristic basal-to-apical gradient, while the pleural line remains regular and consolidations are generally absent. The double-lung-point sign, defined as an abrupt transition between an interstitial pattern in the dependent lung regions and relatively spared upper fields, may support the diagnosis, although it is not consistently present and should not be considered essential for diagnosing TTN [257]. Moreover, the double-lung-point sign may also occur in other neonatal disorders, including respiratory distress syndrome(RDS), meconium aspiration syndrome (MAS), and pneumonia; therefore, it should always be interpreted in conjunction with the clinical context and the overall LUS pattern [258]. Meta-analyses have shown that LUS has high diagnostic performance for TTN, although reported sensitivity varies according to the diagnostic criteria, the reference standard, and whether the double-lung-point sign is assessed alone or together with B-lines [259,260,261].

By contrast, neonatal RDS is characterized by diffuse loss of aeration, compact or coalescent B-lines, an irregular or thickened pleural line, and, in more severe cases, small peripheral consolidations or a homogeneous “white-lung” pattern [4].

These findings reflect the progressive reduction in pulmonary aeration associated with surfactant deficiency and may be quantified using semiquantitative LUS scores [262]. The LUS scores correlate with oxygenation impairment and have been investigated as a tool for identifying infants who are likely to require surfactant therapy [262]. Firstly, the LUS score employed by Brat et al. is a neonatal-adapted, quantitative scoring system in which multiple predefined lung areas are assessed and assigned points according to the severity of LUS patterns (ranging from normal aeration to subpleural consolidations), yielding a total score that increases with worsening lung aeration and parenchymal involvement [263]. In neonates treated with continuous positive airway pressure (CPAP), Brat et al. demonstrated that their LUS score was significantly correlated with multiple indices of oxygenation—including the transcutaneous partial pressure of oxygen to fraction of inspired oxygen ratio (Po_2_/FiO_2_), alveolar–arterial gradient, oxygenation index, and arterial-to-alveolar ratio—independent of gestational age [263]. Furthermore, their LUS score showed good diagnostic accuracy for predicting the need for surfactant administration, particularly in preterm infants born before 34 weeks of gestation (area under the curve [AUC] 0.93; 95% CI 0.86–0.99; *p* < 0.001). In this subgroup, a LUS cutoff of 4 identified all infants who subsequently received surfactant with 100% sensitivity and with moderate specificity (61%).

Subsequent studies have confirmed the predictive value of different neonatal LUS scores, with Raimondi’s quantitative score showing an area under the curve of approximately 0.87 for predicting surfactant requirement in late-preterm and term neonates, and demonstrating comparable accuracy to that previously reported in more immature preterm populations [264].

Accordingly, serial LUS may complement clinical examination, oxygen requirement, blood-gas analysis, and respiratory-support parameters when distinguishing predominantly fluid-clearance disorders such as TTN from surfactant-deficiency disease and when assessing the progression of RDS. In selected infants with RDS, LUS may also support earlier and more individualized surfactant administration, potentially reducing reliance on delayed radiography or oxygen-based criteria alone [265]. However, LUS scores should be considered decision-support tools rather than standalone treatment thresholds, because their performance is influenced by gestational age, timing of examination, respiratory support, scoring system, operator expertise, and the clinical criteria used to administer surfactant [266].

In a multicenter prospective diagnostic accuracy study, Hoshino et al. demonstrated that LUS aeration scores accurately predict the need for surfactant administration in both mechanically ventilated and non-ventilated preterm infants with RDS, with excellent discrimination (AUC 0.975 and 0.915, respectively) and strong correlation with radiographic severity and post-treatment oxygenation improvement [267].

Notably, a recent randomized pilot study did not demonstrate a significant difference between LUS-directed and conventional surfactant treatment, underscoring the need for further prospective validation before LUS can replace integrated clinical decision-making [268].

In MAS, LUS typically reveals a heterogeneous alveolar–interstitial pattern, with coalescent or irregular B-lines, pleural-line abnormalities, and multiple subpleural consolidations containing air bronchograms; atelectasis and, less frequently, pleural effusion may be observed in more severe cases [269].

The distribution of these findings is variable and may change over time, reflecting the patchy and dynamic deposition of meconium within the lungs. LUS may therefore assist in the early bedside diagnosis, severity assessment, and monitoring of MAS while limiting exposure to ionizing radiation [270].

## 8. Clinically Oriented LUS Flowcharts for the Clinician

To complement the description of sonographic signs and technical aspects, the following flowcharts provide a clinically oriented framework for integrating LUS findings into bedside decision-making (Figure 13). Rather than focusing on predefined diagnoses, these algorithms start from common pediatric clinical presentations and guide the clinician through the interpretation of LUS findings and the need for further imaging or intervention. Three representative scenarios are proposed: the child with cough, fever, wheeze, or increased work of breathing; the child with acute respiratory distress; and the patient with suspected pleural disease or peripheral lung lesion. These flowcharts are intended as practical educational tools to support, rather than replace, clinical judgment and established diagnostic pathways. They are expert-derived and were developed by authors of this manuscript who are members of a national panel of Italian pediatric LUS experts, based on the available literature, current consensus recommendations, and clinical experience [33].

## 9. Strengths and Constraints of LUS in Pediatric Practice

A key advantage of LUS in pediatrics is the avoidance of ionizing radiation, enabling safe repeated assessments [38]. Standard CXR, while inexpensive and widely available, has limited sensitivity for differentiating alveolar from interstitial pneumonia, is subject to interobserver variability, and still exposes children to radiation [271,272,273,274,275]. Meta-analyses confirm that LUS outperforms CXR for sensitivity while maintaining similar specificity in diagnosing pediatric pneumonia [53,276,277].

LUS also enables early recognition of small consolidations, pleural effusions and PTX often occult to CXR and provides a safe tool for follow-up, limiting repeated radiation exposure [278]. On a higher level, CT offers detailed evaluation of pleura and lung but is inappropriate for routine use given its radiation burden [34]. To prevent unnecessary radiation exposure, guidelines recommend against routine or indiscriminate use of ionizing imaging in children unless there is a clear medical need [279,280].

Although LUS has demonstrated substantial diagnostic potential in pediatric care, its interpretation remains constrained by significant limitations, including high operator dependency and the intrinsic technical constraints of the modality.

In fact, LUS cannot replace CXR in routine pediatric practice, nor CT imaging, especially when a comprehensive evaluation of mediastinal, central airway, or deep parenchymal structures is required. Higher-level cross-sectional imaging remains essential for conditions distant from the pleura (e.g., congenital pulmonary airway malformations, bullae, pulmonary or subcutaneous emphysema, interstitial emphysema, quantification of PTX, deep mediastinal lesions). In cases where clinical presentation does not align with sonographic findings or symptoms persist, further imaging should be pursued to avoid misdiagnosis [140,281].

To date, studies comparing LUS with chest CT—considered the gold standard for lung imaging—remain limited and often of low methodological quality. This raises questions regarding the diagnostic accuracy of LUS for pneumonia, particularly in the detection of small or deep lesions not in contact with the pleura [282]. These considerations highlight that LUS should not be regarded as a standalone diagnostic modality, but rather as a complementary tool to be integrated with clinical, laboratory, and other imaging findings.

According to the American Academy of Pediatrics, a major limitation of LUS is its significant operator dependency. The accuracy and reproducibility of the technique are directly correlated with the examiner’s training and experience [65]. Adult studies suggest that LUS expertise can be acquired relatively quickly, but still requires a minimum learning curve [235,283]. Systematic reviews in pediatrics confirm that variations in sonographer expertise and the severity of the disease are major sources of heterogeneity and misdiagnosis [101,284]. To ensure reliable practice, the scientific community emphasizes the need to clearly define professional roles, establish structured training pathways, and determine the number of supervised procedures required before independent use [59].

A limitation of the current evidence base is its uneven pediatric representation: some LUS applications are supported by dedicated pediatric prospective or observational studies, whereas others still rely substantially on adult-derived data, secondary literature, or expert consensus. Accordingly, diagnostic-performance estimates and quantitative thresholds should be interpreted according to the population in which they were originally validated.

The adoption of LUS in pediatrics faces several challenges, primarily the absence of structured training in the early stages of residency programs; in fact, few pediatric residency programs provide formal LUS curricula compared with other specialties [285,286]. Evidence shows that structured training improves both diagnostic accuracy and inter-observer reliability. As a matter of fact, a dedicated LUS training program enhanced pneumonia diagnosis and allowed differentiation of bacterial from viral patterns, with strong agreement between trained physicians and radiologists [287]. Surveys of European neonatal units highlight the lack of standardized education and international guidelines as major obstacles to widespread implementation, while formal curricula and consensus recommendations are seen as crucial to improve reliability [288,289].

To operationalize standardized competency in LUS, Musolino et al. [33] engineered a rigorous, longitudinal core curriculum for pediatric LUS validated through a modified, three-round Delphi consensus process among Italian academic experts, which yielded a consensus threshold of 80% agreement on pedagogical components. Rooted structurally in the I-AIM (Indication, Acquisition, Interpretation, Medical Decision-Making) framework and calibrated against Bloom’s revised taxonomy of learning domains, this educational paradigm delineates a strict four-tiered proficiency pyramid: Foundational, Secondary, Advanced, and Masterclass. Progression through these milestones governs specific, escalating clinical privileges. To overcome the documented limitations of isolated theoretical or asynchronous evaluations, the experts mandated a dual-component summative assessment strategy: standardized written examinations designed to objectively evaluate cognitive sub-competencies in ultrasound physics, knobology, and artifact semiotics (e.g., A/B-lines, consolidations), combined with Objective Structured Clinical Examinations (OSCE) to systematically measure real-time clinical integration and diagnostic synthesis. Concurrently, the curriculum enforces a highly regulated, hands-on practical pathway requiring a minimum of 30 supervised scans within six months for Foundational accreditation, scaling up to 60 supervised procedures for the Secondary and Advanced tiers. Longitudinal validation is strictly formalized through digital logbook archiving via cloud storage and tutor verification. Furthermore, Advanced candidates face an objective five-station national commission appraisal requiring a 60% cumulative passing score distributed across predefined domains—specifically weighting examination appropriateness 20%, technical execution accuracy 20%, final diagnostic correlation 40%, and multimodal radiological concordance 20%—culminating in a time-limited certification subject to mandatory independent recertification every four years to maintain clinical privileges and mitigate operator-dependent skill decay [33].

As an aside, image storage should always be granted, as it is crucial for consultation, quality assurance, education, and medicolegal purposes [194].

More broadly, LUS is limited in detecting deep parenchymal or central airway lesions not in contact with the pleura, and sonographic features of different pulmonary diseases often overlap, reducing specificity [16,146]. Another intrinsic limitation is the reduced ability to visualize mediastinal abnormalities using transthoracic scanning, especially in ossified rib cages and patients older than 12 months [108,290]. Finally, image quality may also be compromised in the presence of subcutaneous emphysema, obesity, or chest wall deformities [16,146].

In addition, patient cooperation may represent a practical limitation, particularly in the first years of life. In newborns, contact with the probe and gel may trigger distress or crying, potentially compromising image acquisition. Similarly, in older infants and children, anxiety and lack of cooperation may reduce examination feasibility and quality. Non-pharmacological strategies may help mitigate distress, including non-nutritive sucking in neonates, which has been shown to reduce pain-related behaviors [291], as well as physical comfort measures and age-appropriate distraction techniques (e.g., toys, music, or interactive activities), which can improve compliance across pediatric age groups [292].

## 10. Conclusions

LUS is progressively evolving from a niche diagnostic tool to a valuable component of contemporary pediatric respiratory assessment. Its unique advantages, e.g., radioprotection, bedside availability, and capacity for real-time monitoring, make it a particularly valuable imaging tool in children. This narrative review highlights how LUS can support the diagnosis and management of a wide range of pediatric pulmonary conditions, including infectious, traumatic, and cardiogenic processes. Nonetheless, intrinsic technical and anatomical considerations, operator dependency, and limited visualization of deep or mediastinal structures have to be acknowledged as significant limitations. Strengthening structured training pathways, standardizing scanning protocols, and integrating LUS into pediatric curricula will be crucial steps for ensuring consistent, reproducible application. As technological innovations continue to refine image quality and automated analysis, LUS has the potential to become an increasingly central tool in pediatric care, complementing traditional radiologic imaging.

## Figures and Tables

**Figure 1:**
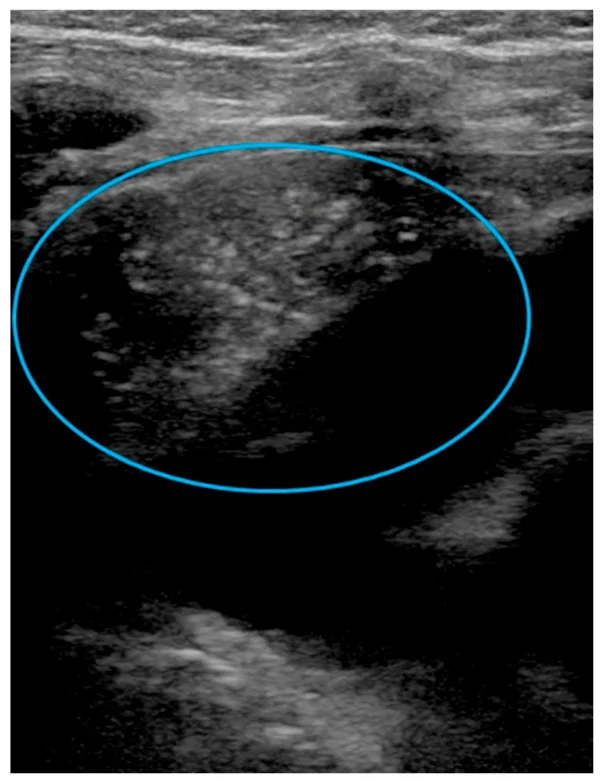
Below the chest wall, the thymus is visible. It has a hypoechoic structure with bright internal echoes and punctate foci corresponding to septa and vessels. The ultrasound image of the thymus is highlighted by the blue circle.

**Figure 2:**
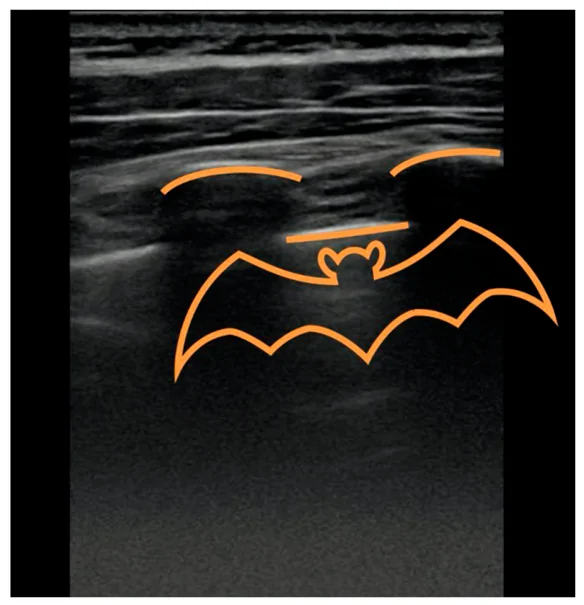
The “bat” sign.

**Figure 3:**
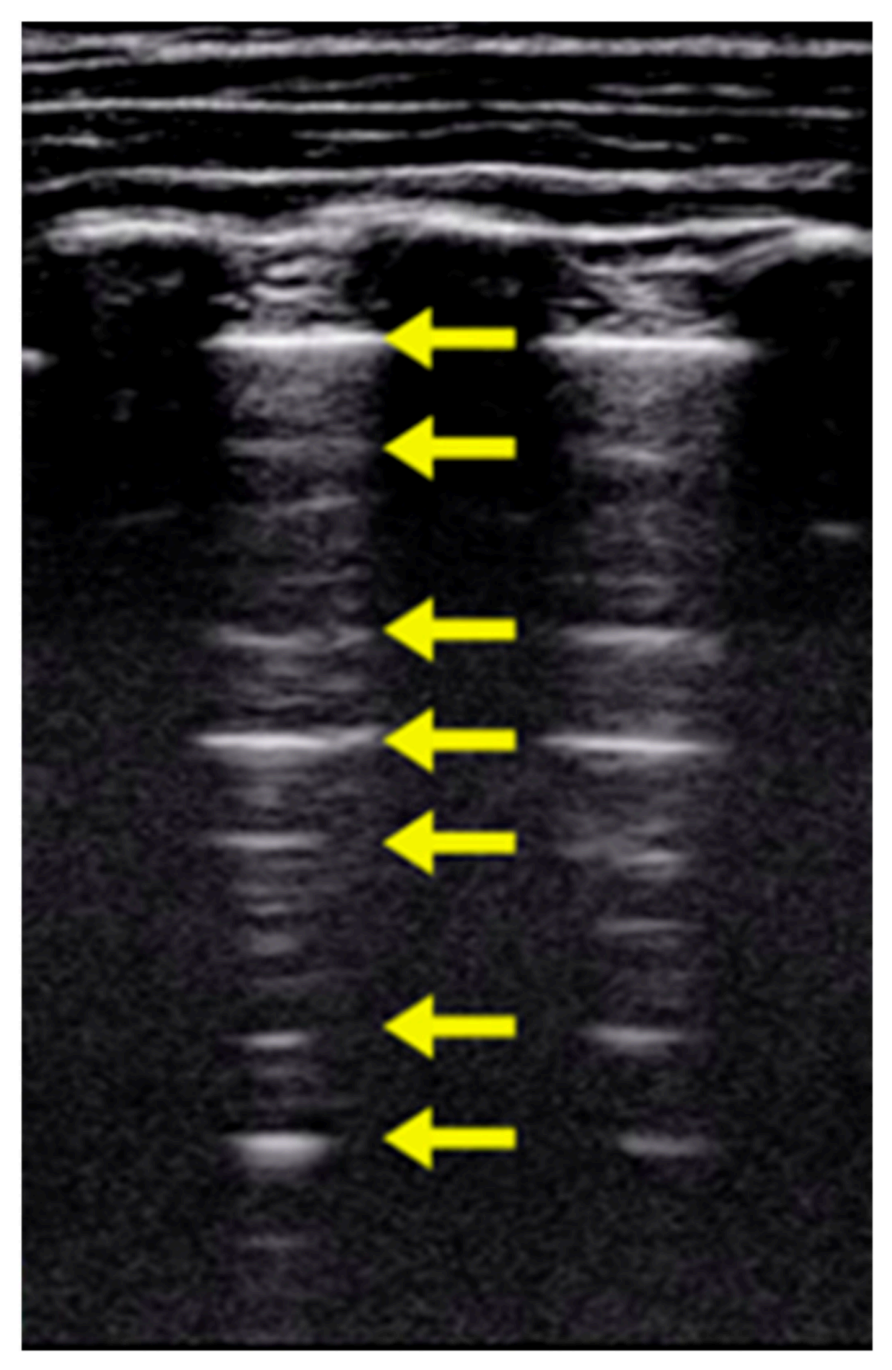
The yellow arrows highlight A-lines.

**Figure 4:**
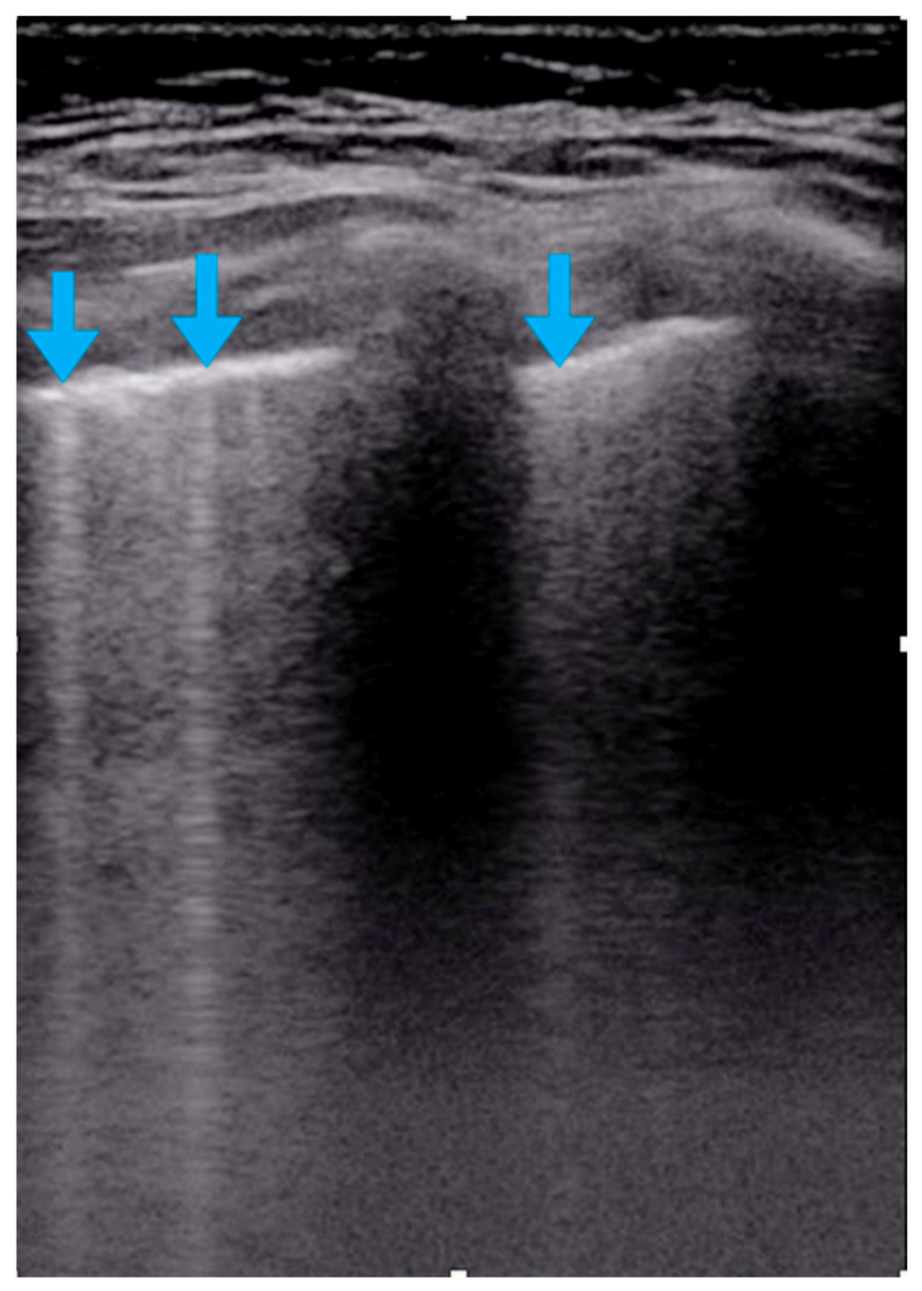
The blue arrows highlight isolated, non-confluent B-lines.

**Figure 5:**
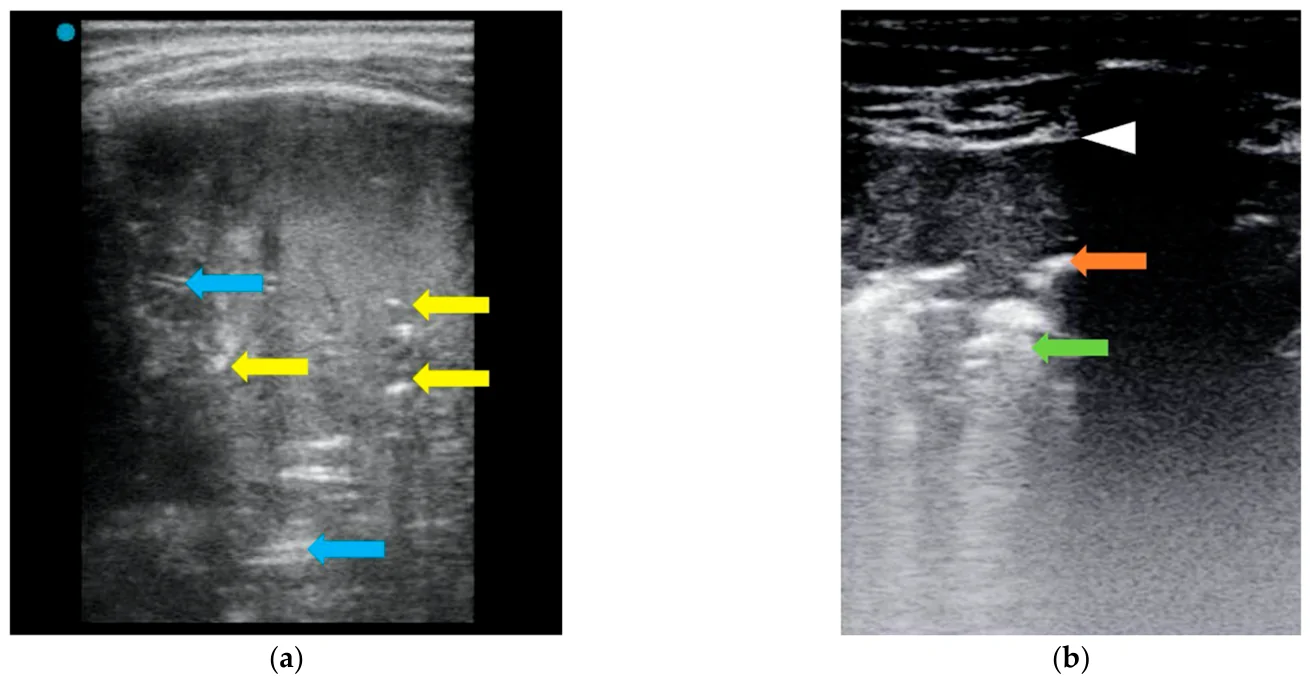
Main features of pneumonia consolidations. (**a**) A transverse lung scan with a linear probe shows a large lung consolidation with absence of A-lines and a tissue-like appearance. Yellow arrows highlight air bronchograms, while blue arrows indicate fluid bronchograms within the consolidation. (**b**) A longitudinal lung scan with a linear probe highlights a subpleural consolidation. The white arrowhead points to the overlying pleural line, which has an irregular, partially hypoechoic appearance. The orange and green arrows indicate the shred sign.

**Figure 6:**
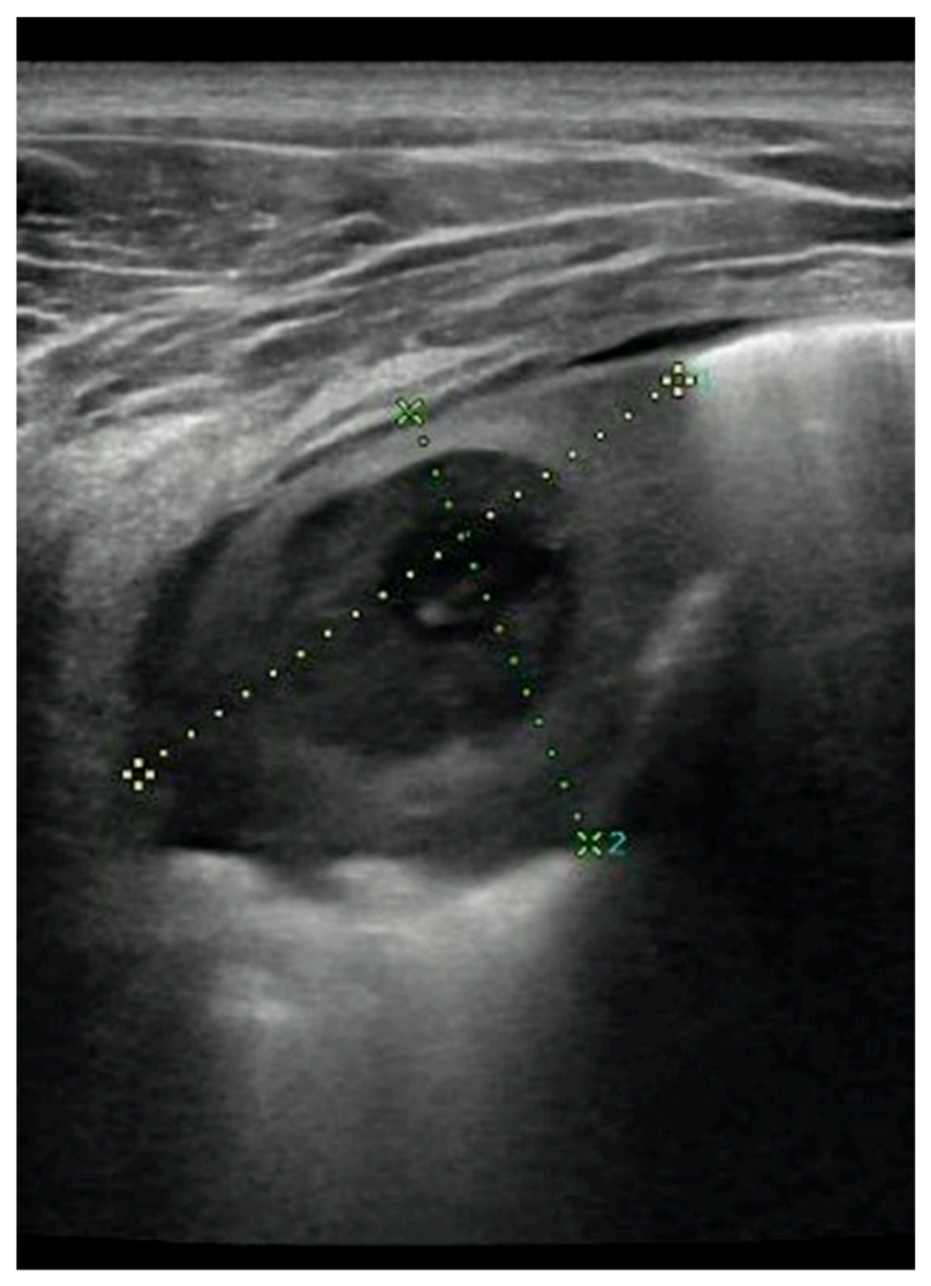
A subpleural lung abscess presents with a rounded shape and a thick peripheral, irregular wall consisting of adjacent collapsed lung parenchyma.

**Figure 7:**
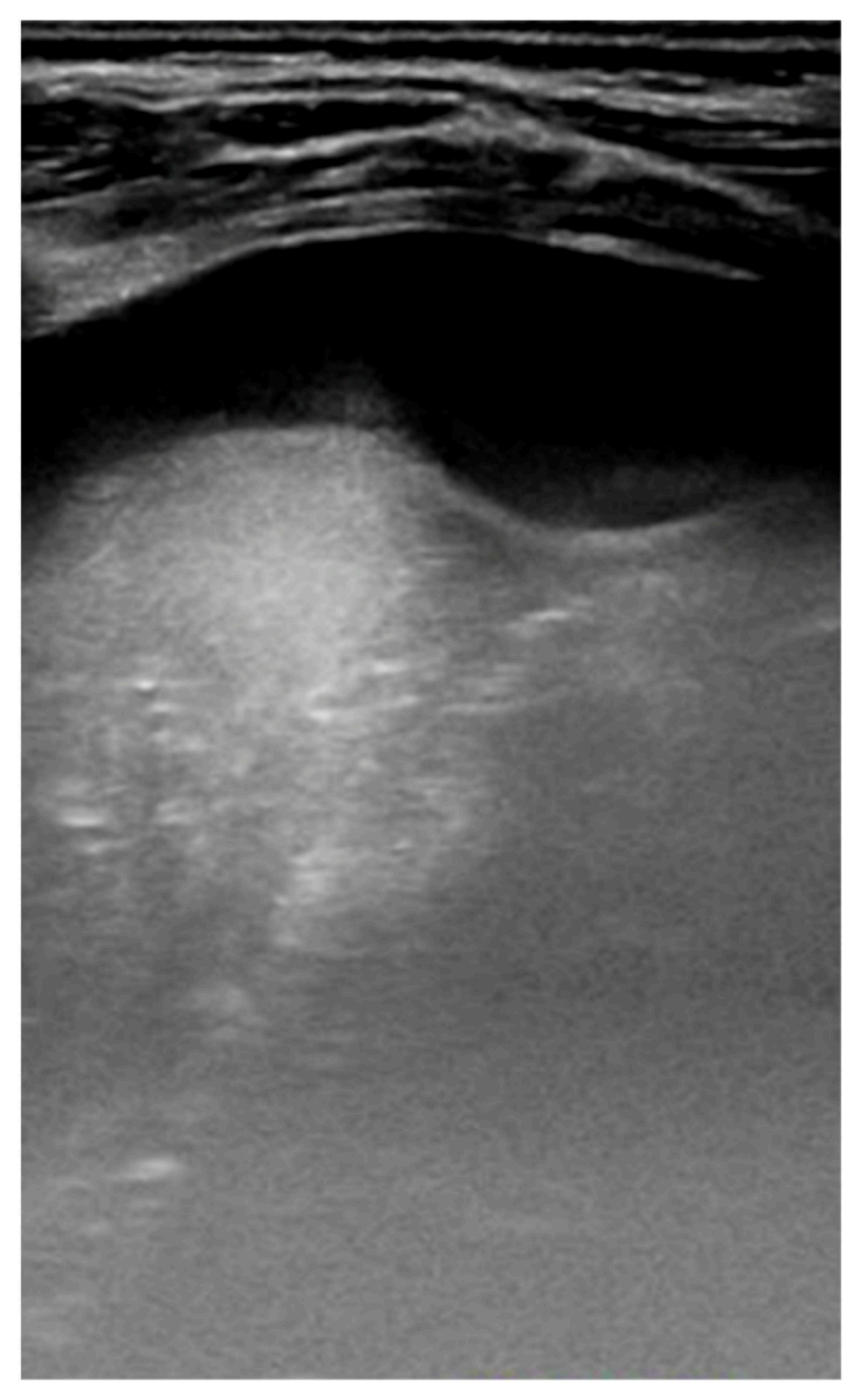
Atelectasis with static bronchograms, within a pleural effusion.

**Figure 8:**
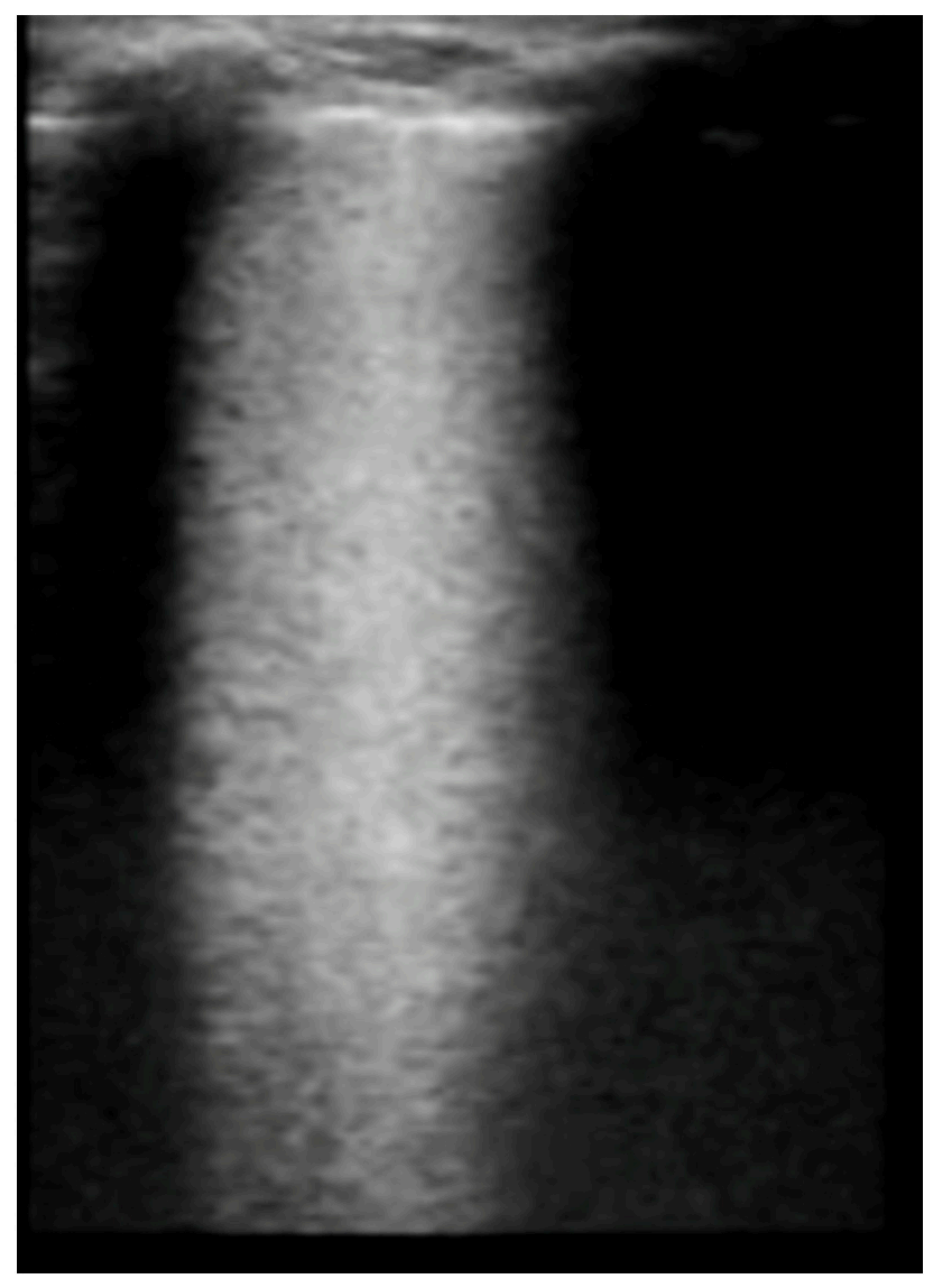
Coalescent B-lines as shown in bronchiolitis.

**Figure 9:**
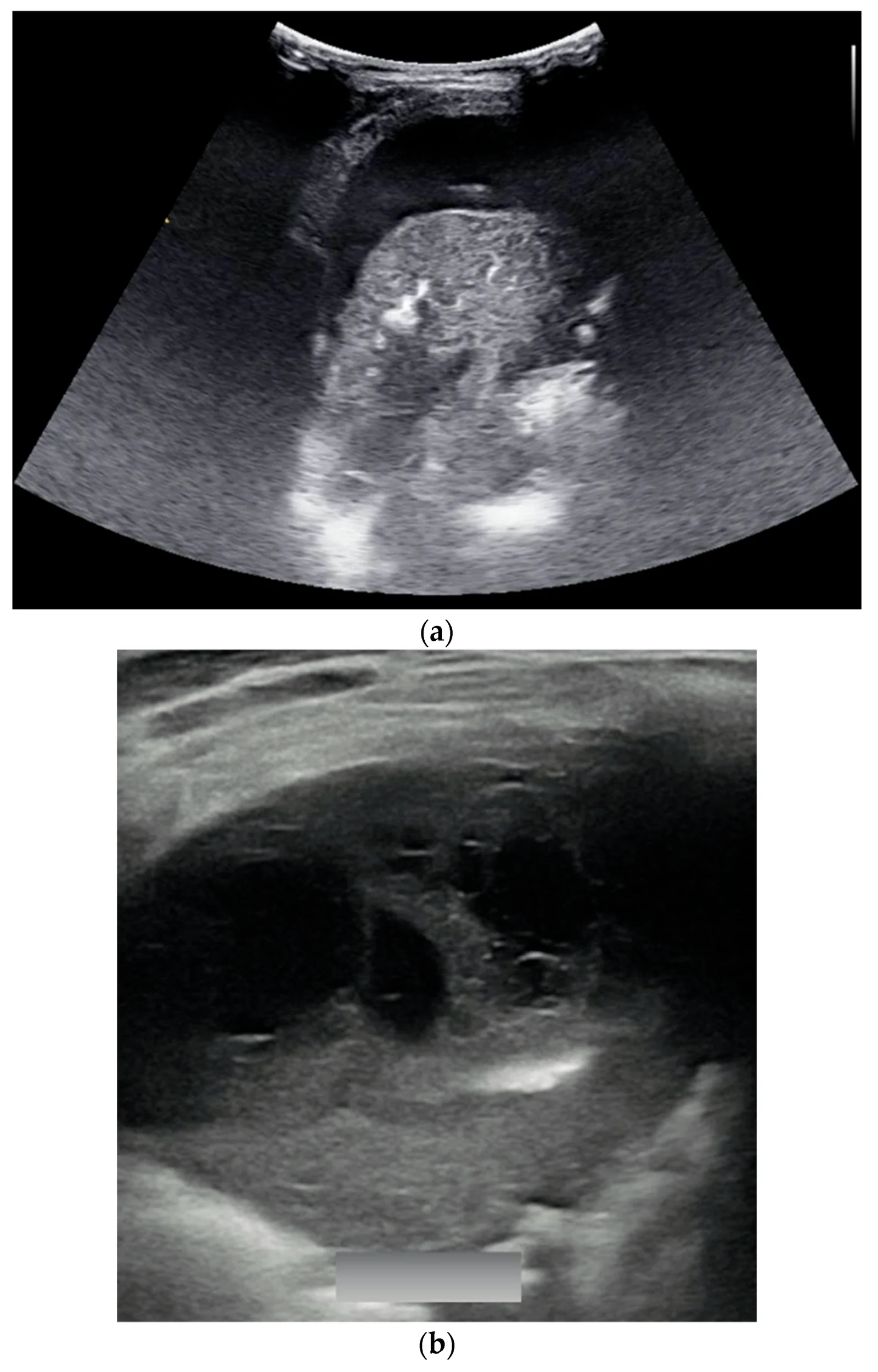
(**a**) There is an infectious exudate associated with an underlying consolidation, and a few initial fibrin elements can be seen in the exudate. Thickened pleura can be seen overlying the exudate. (**b**) Complicated pleural empyema with numerous septations.

**Figure 10:**
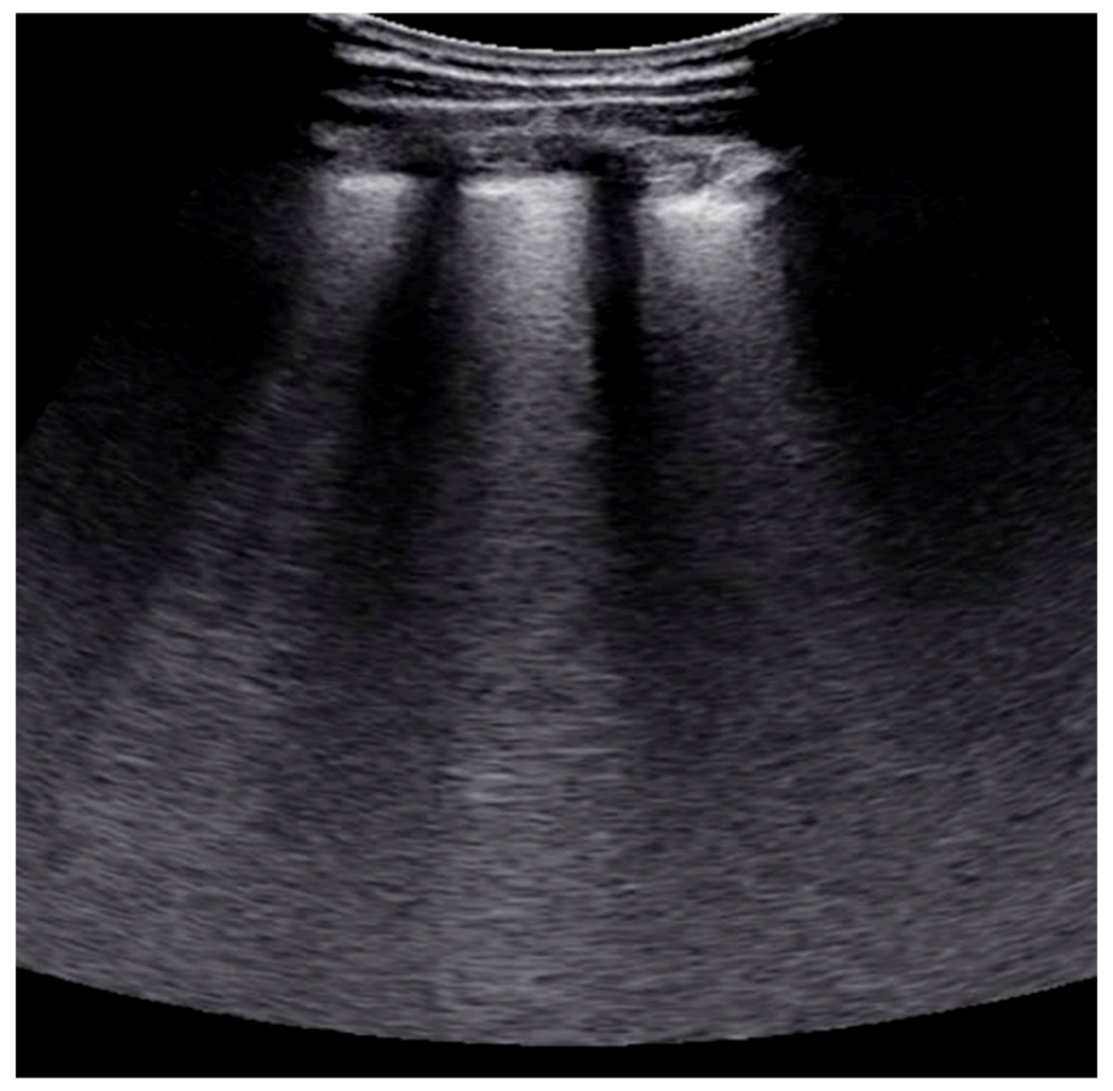
Pulmonary edema.

**Figure 11:**
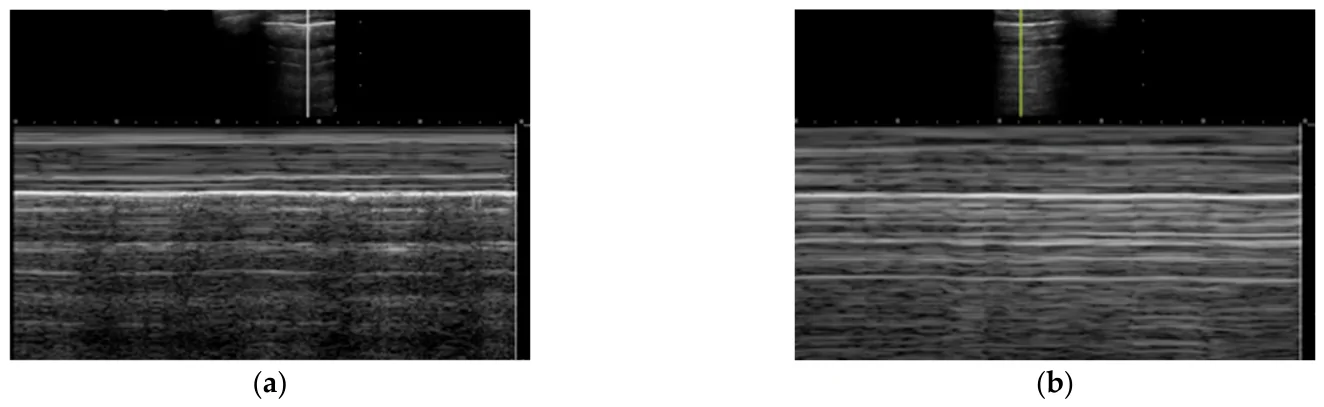
(**a**) a physiological lung M-mode scan, with the characteristic “seashore sign”, characterized by a sandy pattern. (**b**) a “barcode sign” in M-mode, confirming absent pleural sliding and confirming pneumothorax.

**Figure 12:**
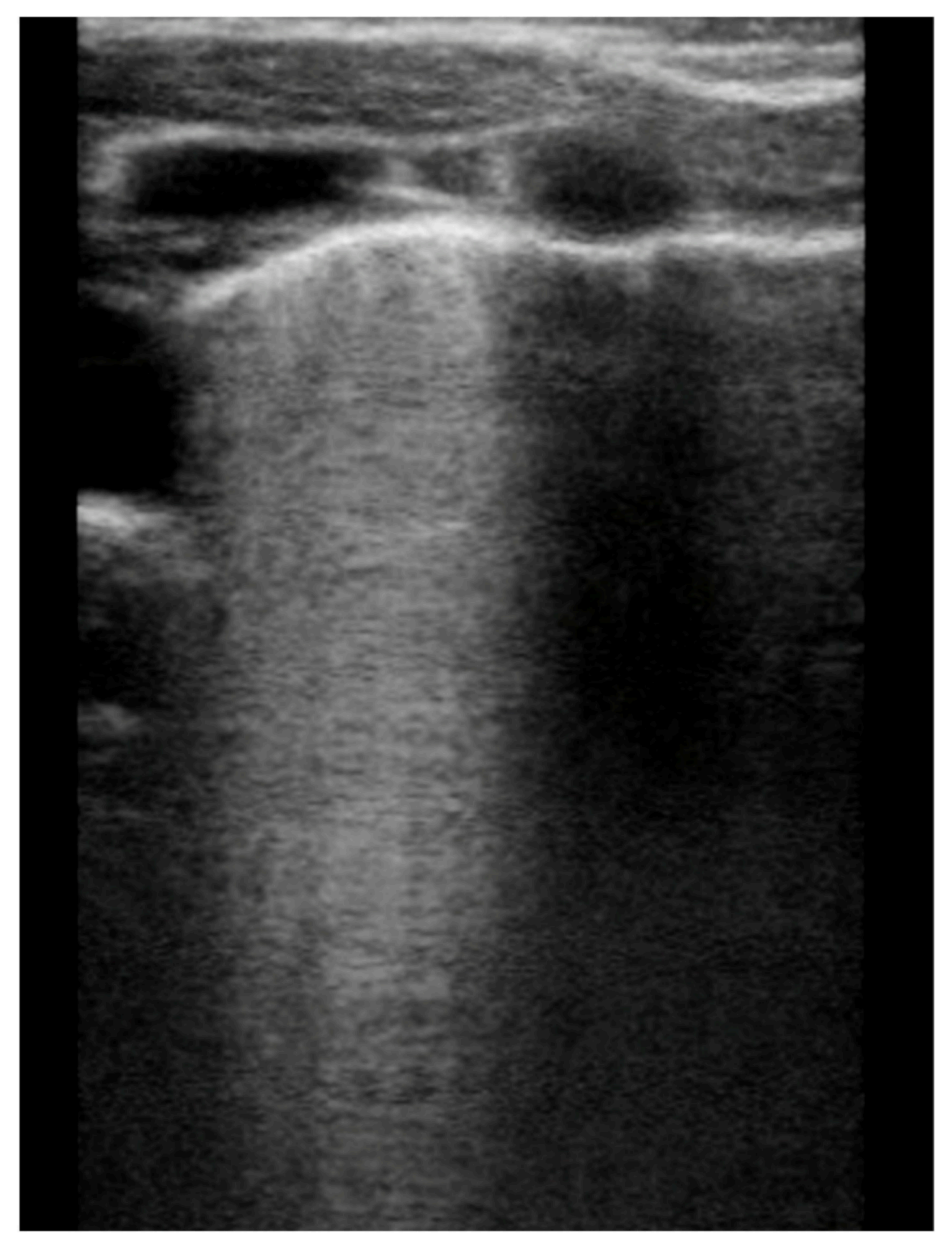
Pulmonary contusion.

**Figure 13:**
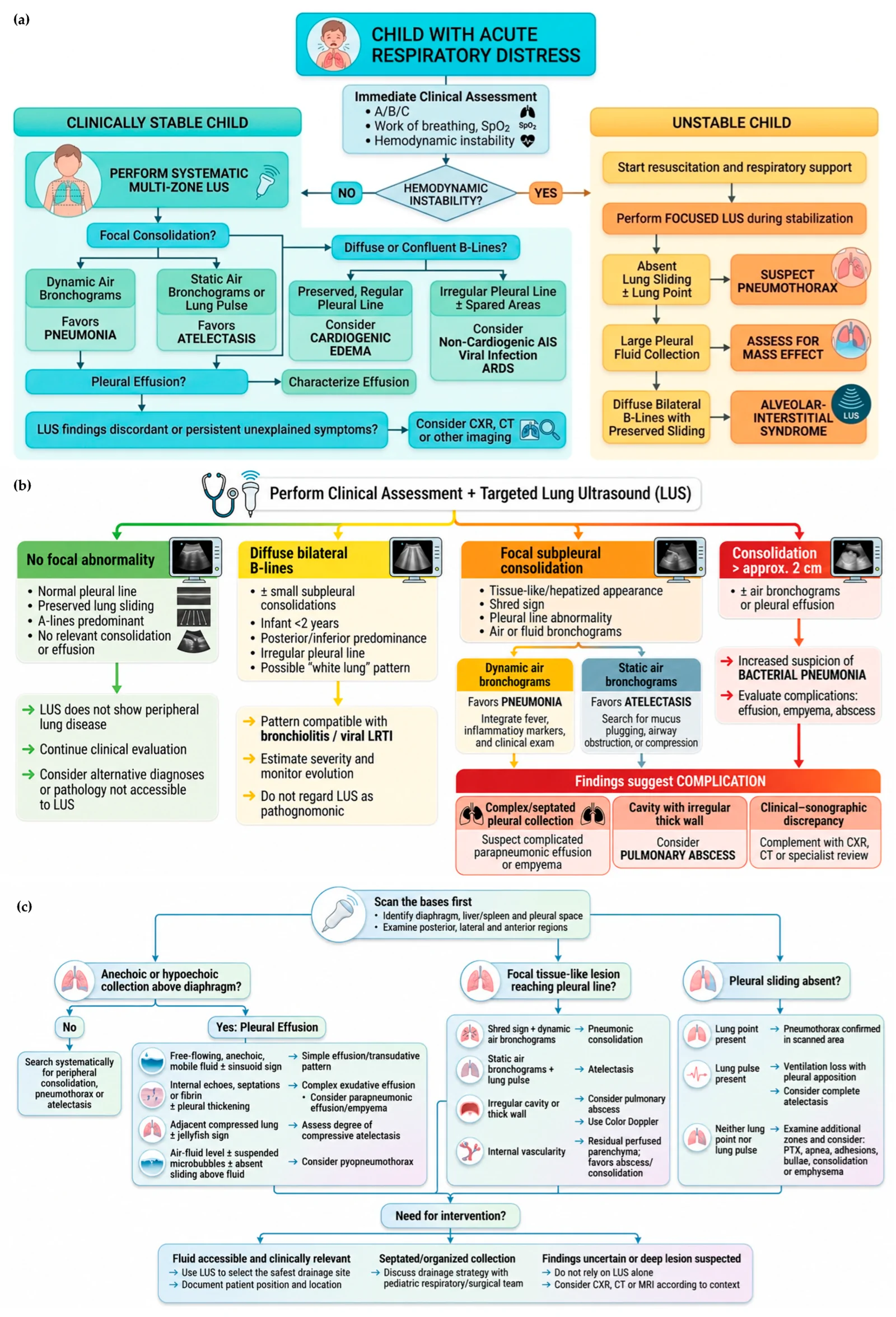
Clinically oriented LUS decision-making flowcharts for common pediatric presentations: (**a**) acute respiratory distress; (**b**) cough, fever, wheeze, or increased work of breathing; and (**c**) suspected pleural disease or peripheral lung lesion. The algorithms integrate clinical assessment, LUS findings, and indications for further imaging or intervention.

**Table 1:** Overview of probe types, features and applications.

Probe Type	Frequency (MHz)	Footprint	Depth (cm)	Primary Applications	References
Linear	6–12	Rectangular, flat	1–4 (superficial)	Neonatal/pediatric LUS (preferred for anterior/lateral zones, high-res pleural line, B-lines, small infants)	[52,53,60,63,65]
Microconvex	5–8	Small, curved	2–8 (intermediate)	Neonatal LUS (good for intercostal spaces, all zones, small chest wall)	[60,63,65]
Convex	3–5	Large, curved	4–15 (deep)	Older children, deeper lung	[51,52,58,59,64,65,66]
Phased-array	2.5–5	Small, square/sector	4–20	Pediatric LUS (especially between ribs, posterior/basal zones, older children)	[52,53,59,60,66,67,68]

**Table 2:** Characteristics of scanning protocols.

Scanning Approach	Chest Division and Anatomical Landmarks	Main Population	References
Twelve-zone protocol	Thorax divided by anterior and posterior axillary lines into 6 zones per hemithorax; 12 total: anterior, lateral and posterior regions, each divided into upper and lower areas by two axial lines: one about 1 cm above the nipples and the further above the diaphragm	Pediatric patients	[59]
Assessment of pulmonary aeration (for the computation of the LUS aeration score) according to the age of the patient	A 12-region approach (6 per hemithorax) is recommended in adults and children > 1-year-old: anterior, lateral, and posterior fields are identified by sternum, anterior, and posterior axillary lines; each field is divided into superior and inferior regions. In infants ≤ 1-year-old and neonates after the first 24–48 h of life, a 5-region per hemithorax approach with a single lateral region is recommended. In neonates (particularly if preterm) for the first 24–48 h, a simplified approach limited to 3 regions per hemithorax (upper and lower anterior field and one lateral field) is advised	ICU patients, divided by age	[79]
Six-zone pediatric approach	Longitudinal intercostal scanning of the anterior lung fields along the parasternal, midclavicular, and anterior axillary lines, from the diaphragmatic to the apical regions, together with mandatory posterior evaluation along the posterior axillary, medial, and paravertebral lines; suprasternal and supra-/infraclavicular views are used to assess the lung apices	Pediatric patients	[29]
AAP neonatal six-zone protocol	3 zones per hemithorax; 6 total: an upper anterior zone (from the parasternal to the midclavicular line) and a lower anterior zone (from the midclavicular to the anterior axillary line), plus a lateral zone along the anterior-to-mid axillary line, all scanned longitudinally with the infant supine.	NICU patients/newborns	[65]
Fourteen-zone Soldati protocol	7 zones per hemithorax; 14 total: 3 posterior, 2 lateral and 2 anterior. Posterior scans: upper, middle and basal regions along the paravertebral line; lateral scans: upper and basal regions on the midaxillary line; anterior scans: upper and basal regions on the midclavicular line. The internipple line separates upper from basal anterior/lateral areas; the basal posterior scan is performed above the curtain sign	Adult-derived; proposed in pediatric patients	[80]

**Table 3:** Artifacts and signs in lung ultrasound.

Artifacts and Signs	Description	Where to Find Them
A-lines	Horizontal hyperechoic lines, parallel and equidistant from the pleura, produced by reverberation artifacts when ultrasound waves reflect repeatedly between strong interfaces like the chest wall and a normal aerated lung	Normal lung Pneumothorax
B-lines	Vertical hyperechoic artifacts arising from the pleural line and extending toward the far field, typically erasing the underlying A-lines. They are associated with altered peripheral lung aeration and increased tissue–air acoustic interactions	Pulmonary edema Interstitial phlogosis (including pneumonia) ARDS Interstitial lung disease/fibrosis (Isolated or limited B-lines may also occur under physiological conditions)
Scattered B-lines	Discontinuous, non-uniformly distributed B-lines across scanned zones, with adjacent intercostal spaces showing preserved A-lines; may be associated with mild-to-moderate or early interstitial disease	
Compact B-lines	Multiple, closely spaced, densely aggregated B-lines within a single intercostal space or scanning zone, with markedly reduced visibility of intervening A-lines, suggesting more pronounced regional interstitial fluid	
Coalescent B-lines	Numerous, tightly packed B-lines that partially merge, forming nearly continuous vertical bands from the pleura, suggesting more severe interstitial involvement and increased extravascular lung water	
Barcode/stratosphere sign	In M-mode, a series of parallel horizontal lines corresponding to absence of pleural sliding in B-mode	Pneumothorax
Bat sign	In longitudinal scans, a shape formed by the profile of two ribs and the pleural line between them	Normal lung Not visible in subcutaneous emphysema
C-lines	Vertical artifacts arising from the tissue–air interface located within or along the edges of a consolidated lung, produced by the distortion of airspace geometry in areas of pre-consolidation	Pneumonia
Curtain sign	The air in the costophrenic recess casting a hyperechoic “air curtain” over the recess, obscuring the outline of the lateral diaphragm and moving with respiratory acts	Normal lung
Dynamic air bronchogram	Hyperechoic foci (the air within bronchi) displaying synchronous motion with inspiration and expiration	Pneumonia Excludes atelectasis
E-lines	Vertical hyperechoic artifacts that can be mistaken for B-lines but do not originate at the pleural line and show no respiratory movement	Subcutaneous emphysema
Fluid bronchogram	Anechoic, tubular structures within the lung parenchyma, without blood flow in Doppler study	Pneumonia
Fluid color sign	In color Doppler, a color signal within a collection of pleural fluid moving in synchrony with respiratory or cardiac movement	Pleural effusion Absent in pleural thickening or solid tissue
Jellyfish sign	Consolidated or atelectatic lung tissue moving within an anechoic collection	Large pleural effusion
Lung point	Transition zone between the margin of a pneumothorax, where the pleural layers are separated, and the normally aerated lung with intact pleural apposition	Pneumothorax (not visible in complete pneumothorax)
Hepatization/tissue-like sign	Hypoechoic region (compared with the surrounding lung) due to the presence of fluid in the lung, with echogenicity similar to that of the liver	Pneumonia Atelectasis Pulmonary contusion
Pleural sliding	A pleural line movement associated with respiration acts. This motion is easily seen as a shimmering or “ants marching on a log” appearance	Normal lung Absent in pulmonary adhesions, atelectasis, pneumothorax and apnea
Pulse sign	A periodic vertical motion synchronous with cardiac pulsations that are transmitted through the pulmonary parenchyma, associated with loss of ventilation	Complete atelectasis Selective intubation Apnea Excludes the possibility of pneumothorax in the examined location
Quad sign	A quadrilateral shape whose four sides are formed by nearly parallel pleural layers, separated by effusion, and two adjacent rib shadows	Pleural effusion
Seashore sign	In M-mode, a sand-like pattern below the “waves” of the pleural line, corresponding to pleural sliding	Normal lung Absent in pneumothorax
Shred sign	An irregular, hyperechoic border that demarcates a lung consolidation from the adjacent aerated parenchyma	Pneumonia
Sinusoid sign	In M-mode a sinusoidal movement of the collapsed but expandable lung in the fluid, synchronized with respiratory acts	Complicated pneumonia with effusion
Spine sign	When fluid is present, it appears as an anechoic triangular space above the diaphragm and allows vertebral bodies or proximal ribs to appear into the thorax	Pleural effusion
Static air bronchogram	Air trapped within collapsed bronchi, with lack of movement throughout the respiratory cycle	Atelectasis
Suspended microbubble sign	Hyperechoic spots with shadows that move randomly with breathing movement within the pleural effusion	Pyopneumothorax

## Data Availability

No new data were created or analyzed in this study. Data sharing is not applicable to this article.

## Abbreviations

The following abbreviations are used in this manuscript:

AIS	Alveolar-interstitial Syndrome
ARDS	Acute Respiratory Distress Syndrome
CXR	Chest X-ray
CAP	Community-acquired Pneumonia
CT	Computed Tomography
ESICM	European Society of Intensive Care
ESPNIC	European Society of Paediatric and Neonatal Intensive Care
IVC	Inferior Vena Cava
LUS	Lung Ultrasound
MAS	Meconium aspiration syndrome
MI	Mechanical Index
PICU	Pediatric Intensive Care Unit
PTB	Pulmonary Tuberculosis
PTX	Pneumothorax
TB	Tuberculosis
TTN	Transient tachypnea of the newborn
